# Semi-Automated Analysis of Organelle Movement and Membrane Content: Understanding Rab-Motor Complex Transport Function

**DOI:** 10.1111/j.1600-0854.2011.01283.x

**Published:** 2011-10-11

**Authors:** Alistair N Hume, Miranda S Wilson, Dmitry S Ushakov, Michael A Ferenczi, Miguel C Seabra

**Affiliations:** 1 Molecular Medicine, National Heart and Lung Institute, Imperial College LondonLondon SW7 2AZ, UK; 2 School of Biomedical Sciences, Queen's Medical CentreNottingham NG7 2UH, UK; 3 Instituto Gulbenkian de CiênciaOeiras 2780-156, Portugal; 4 Faculdade de Ciências Médicas, Universidade Nova de LisboaLisboa 1169-056, Portugal

**Keywords:** actin, live-cell imaging, melanophilin, melanosome, microtubule, myosin Va, Rab GTPase

## Abstract

Organelle motility is an essential cellular function that is regulated by molecular motors, and their adaptors and activators. Here we established a new method that allows more direct investigation of the function of these peripheral membrane proteins in organelle motility than is possible by analysis of the organelle movement alone. This method uses multi-channel time-lapse microscopy to record the movement of organelles and associated fluorescent proteins, and automatic organelle tracking, to compare organelle movement parameters with the association of membrane proteins. This approach allowed large-scale, unbiased analysis of the contribution of organelle-associated proteins and cytoskeleton tracks in motility. Using this strategy, we addressed the role of membrane recruitment of Rab GTPases and effectors in organelle dynamics, using the melanosome as a model. We found that Rab27a and Rab32/38 were mainly recruited to sub-populations of slow-moving/static and fast-moving melanosomes, respectively. The correlation of Rab27a recruitment with slow movement/docking was dependent on the effector melanophilin. Meanwhile, using cytoskeleton-disrupting drugs, we observed that this speed:Rab content relationship corresponded to a decreased frequency of microtubule (MT)-based transport and an increased frequency of actin-dependent slow movement/docking. Overall, our data indicate the ability of Rab27a and effector recruitment to switch melanosomes from MT- to actin-based tethering and suggest that a network of Rab signalling may integrate melanosome biogenesis and transport.

Melanosomes are a valuable model for the study of the biology of cytoplasmic organelles. They are specialized pigment/melanin-containing lysosome-related organelles (LROs) present in skin melanocytes (and other pigment cells), whose biogenesis and transport to adjacent keratinocytes are fundamental to mammalian skin and hair pigmentation (1–3). Melanosome biogenesis is proposed to proceed via four morphologically defined maturation stages. Stage I pre-melanosomes are non-pigmented vacuoles, possibly derived from endosomes, which acquire internal striations and mature into stage II organelles. Melanin is then deposited upon these striations, giving rise to partially melanized (stage III) and fully melanized (stage IV) organelles. Stage IV melanosomes are then transferred, via melanocyte dendrites, into numerous adjacent keratinocytes in the basal layer of the epidermis and hair bulb, resulting in normal pigmentation (1,4,5). Current models envisage that mature melanosomes undertake fast long-range transport mediated by conventional kinesin (Kif5b) and cytoplasmic dynein along microtubules (MT) from the cell body into dendrites, where they then transfer to slow short-range myosin Va (MyoVa)-actin-mediated transport or tethering that allows their retention and ultimately transfer to keratinocytes (6–11).

Studies of patients suffering albinism, and coat colour mutant mice, have revealed much of the molecular basis of the biogenesis and transport of melanosomes. Interestingly, these studies also revealed that many of the molecular mechanisms regulating melanosome biogenesis and transport are conserved among LROs present in other cell types, e.g. platelet dense granules, mast cell secretory granules, cytolytic granules in T cells, neutrophil azurophilic granules (12,13). Thus, improved understanding of melanosome biology has broader implication for our understanding of the biology of a range of related organelles.

Several such studies revealed fundamental roles for Rab family small GTPases and their effectors in transport and biogenesis of melanosomes (and other LROs) (14). Rab proteins regulate many aspects of vesicular transport between compartments of the secretory and endocytic pathways in eukaryotes. They perform this function by acting as compartment-specific molecular switches that cycle between active GTP- and inactive GDP-bound conformations. Active Rabs recruit effector proteins to the membrane where they are located, and once there, effectors elicit a biological effect, e.g. vesicle tethering, fusion or movement (15,16). The large size of the mammalian Rab family (>60 members) is thought to reflect the complexity of membrane trafficking pathways required for the production of specialized organelles present in their many functionally specialized cell types (17).

Consistent with this, analysis of the chocolate coat colour mutant mouse revealed a role for Rab38, a non-ubiquitously expressed Rab, in the specialized trafficking pathways that underlie pigmentation (18,19). Specifically, Rab38, with the related isoform Rab32, was shown to regulate melanogenesis by controlling intracellular sorting of two key melanogenic enzymes, tyrosinase and tyrosinase-related protein-1 (Tyrp-1), to melanosomes (20). Meanwhile studies of ashen (ash), leaden (ln) and dilute (dil) coat colour mutant mice, as well as Griscelli and Elijalde syndrome patients, revealed that a complex comprising secretory cell type expressed Rab27-GTP, the effector melanophilin (Mlph), and alternative myosin (MyoVa), tethers mature melanosomes in the peripheral dendrites of melanocytes (21–23). Recently, Rab3GEP, previously identified as a guanine nucleotide exchange factor (GEF) for Rab3a, was revealed to activate Rab27a in melanocytes and thereby regulates melanosome distribution via assembly of the Rab27a–Mlph–MyoVa complex (24).

The functions of other Rab proteins have also been linked with melanosomes. For instance, ubiquitous Rab7 is suggested to regulate MT-dependent movement of immature melanosomes via the effector Rab7-interacting lysosomal protein (RILP), which was previously found to modulate cytoplasmic dynein activity (25). This suggested that MT- and actin-dependent melanosome transport steps are regulated by Rab7 and Rab27a, respectively. Ubiquitous Rab8 has also been suggested to regulate melanosome movement along actin filaments (26). Finally, Rab3a, implicated in exocytosis in the nervous system, was reported to associate with purified melanosomes and was suggested to regulate their targeting to the plasma membrane (27).

Previous studies of the molecular mechanisms of melanosome transport in melanocytes and retinal pigment epithelium (RPE) cells used time-lapse microscopy to record movement of melanosomes in mutant cells, and manual tracking to compare this with movements seen in wild-type control cells (9,28,29). Using this approach, the role of the absent protein was inferred from the differences in melanosome movement between mutant and control cells. Here we extend these studies by establishing a new method that allowed us to directly correlate the movement parameters with Rab/effector recruitment status of populations of melanosome within the same cell. Thus, we were able to examine the role of these proteins in melanosome transport more directly than previously and in a more unbiased/quantitative manner.

## Results

### Rab27a associates with a subset of melanosomes and melanin-free cytoplasmic vesicles in living melanocytes

**Figure 1:**
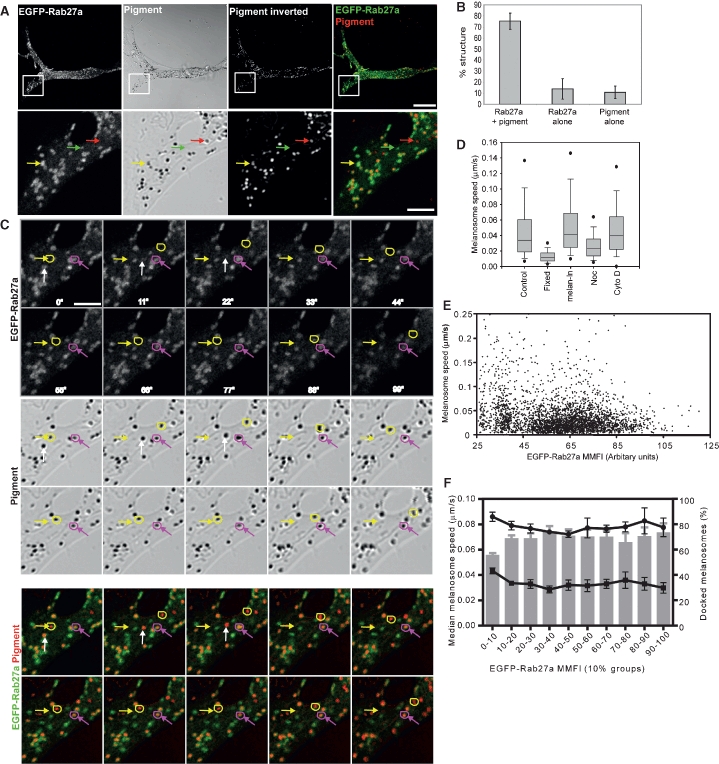
Rab27a-positive melanosomes move more slowly than do the Rab27a-negative melanosomes Rab27a-null melanocytes (melan-ash) were transfected with EGFP-Rab27a and imaged alive using a confocal microscope to simultaneously record the movement of fluorescent protein and melanosomes/pigment as described in *Materials and Methods*. A) The upper panels show an overview of the EGFP-Rab27a expressing melan-ash cell (lacks endogenous Rab27a) used for time-lapse recording. Note that the expression of EGFP-Rab27a results in rescue of melanosome distribution to peripheral dendrites. The white box indicates the area of cytoplasm used for the time-lapse recording. The lower panels display a single frame from a time-lapse series (Movie S1; *t* = 0) showing that cells contain EGFP-Rab27a-positive melanosomes, EGFP-Rab27a-negative melanosomes and EGFP-Rab27a-positive vesicles lacking pigment (indicated by yellow, red and green arrows, respectively). In the overlay image, green and red signals correspond to EGFP-Rab27a and pigmented melanosomes. Scale bar = 20 µm for upper and 5 µm for lower panels. B) Bar chart showing the percentage of each type of melanosome and Rab27a-positive structure in 10 different cells. C) Images from a time-lapse series (numbers indicate elapsed time in seconds; Movie S1) showing the movement of pigmented melanosomes and EGFP-Rab27a (red and green, respectively, in colour combine). Yellow and magenta rings indicate the position of EGFP-Rab27a-positive and EGFP-Rab27a-negative melanosomes in each frame, while corresponding coloured arrows show the starting position of each melanosome. Scale bar = 5 µm. The white arrows in the first three frames indicate the apparent dissociation of a melanosome from a cluster of Rab27a-positive structures. D) Box plot showing the distribution of frame-to-frame melanosome speed data recorded in melan-ash cells expressing EGFP-Rab27a (0.1% DMSO-treated control (*n* = 14 820 events), and nocodazole (Noc) (*n* = 15 414 events) and cytochalasin D (Cyto D)-treated (*n* = 8019 events), fixed melan-ink4a (*n* = 26 805 events), and melan-ln cells (*n* = 9620 events) (recordings obtained from four cells for each condition). Horizontal line indicates median speed, outer bars indicate 25th–75th percentile range and circles indicate the 5th–95th percentile range for each population. E) Scatter plot showing the relationship between EGFP-Rab27a MMFI and speed for each movement event in melan-ash cells expressing EGFP-Rab27a (*n* = 3880 events recorded from one representative cell). Spearman *r* = −0.3572 with 95% confidence interval −0.3852 to −0.3286, p < 0.001. F) Movement events (*n* = 19 186 events) of melanosomes in melan-ash cells transfected with EGFP-Rab27a were binned on the basis of EGFP-Rab27a MMFI, and median speed (mean and SEM of data from four cells shown; left-hand *y*-axis) was plotted for each group (line plot squares). Data within each MMFI bin was filtered (see text) and the median speed of motile events (mean and SEM, line plot circles) and the percentage of static events (mean and SEM, bar plot right-hand *y*-axis) are displayed. Significance testing was conducted as described in *Materials and Methods* and is present in *Supporting Information*.

In preliminary experiments in transgene-rescued immortal Rab27a-null (melan-ash) melanocytes (30), using time-lapse confocal microscopy to simultaneously record the movement of melanosomes (using phase contrast) and transiently expressed enhanced green fluorescent protein (EGFP)-Rab27a, we observed three classes of intracellular structures: Rab27a-positive melanosomes (∼75%), Rab27a-negative melanosomes (∼11%) and EGFP-Rab27a-positive vesicular structures that apparently lacked a melanin core (∼14%; Figure 1A,B). Similar observations of the association of Rab27a with small pigment-free vesicles have been reported in RPE cells (31). In general, Rab27a-positive melanosomes appeared more restricted in their movement relative to Rab27a-negative melanosomes (Figure 1C and Movie S1, yellow versus magenta marked melanosomes, respectively). Melanin-free, Rab27a-positive vesicles and tubules were particularly apparent when EGFP-Rab27a expressing cells were examined using total internal reflection fluorescence microscopy (TIRFM; Movie S2, arrows and arrowheads, respectively). Similar patterns of sub-cellular localization were observed in melan-ash cells expressing EGFP-Rab27b (Rab27a and Rab27b share 71% amino acid identity), and wild-type (melan-ink4a) cells expressing either EGFP-Rab27a or EGFP-Rab27b (data not shown).

### A semi-automated method for quantitative analysis of melanosome movement and Rab27a–Mlph–MyoVa complex recruitment

**Figure 2:**
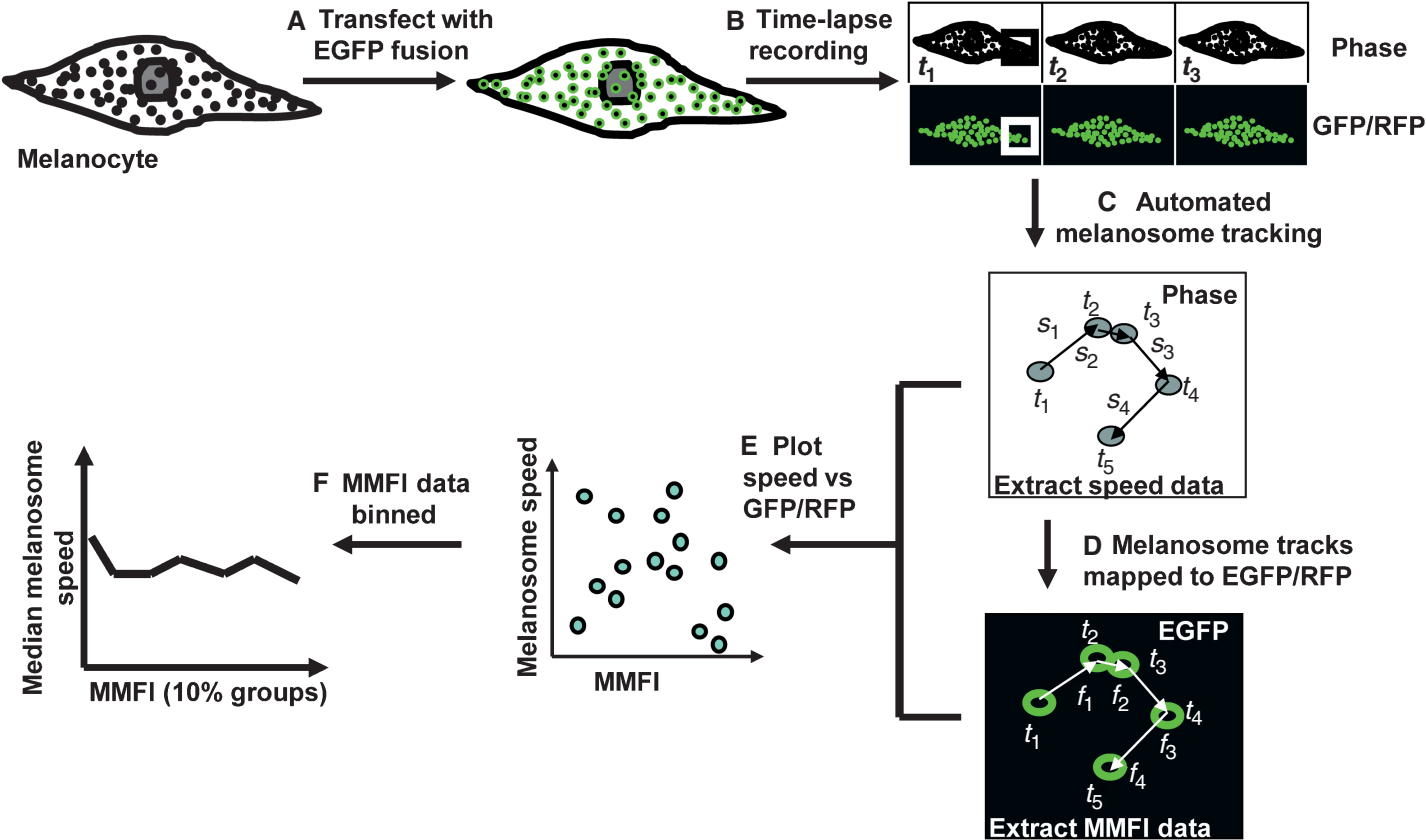
A schematic representation of the melanosome tracking protocol used in this study A) Cultured melanocytes were transiently transfected with melanosome-associated fluorescent fusion proteins. B) Twenty-four or 48 h later, sequences of time-lapse images of transfected cell were recorded using confocal or TIRFM/phase contrast imaging. C) These were imported to Volocity 5.0 image analysis software. Melanosomes were then defined manually on the basis of size and intensity and tracked automatically. Tracks were authenticated visually. The phase contrast box shows an illustration of a melanosome track measured using this method. Track information allowed calculation of the frame–frame melanosome speed, e.g. *s*_1_ is the speed of the melanosome calculated from its displacement from *t*_1_ to *t*_2_. D) Fluorescent protein intensities (*f*_1_, *f*_2_, etc.) associated with tracked frame-to-frame melanosome movement extracted together with speed data (s_1_, s_2_, etc.). E and F) The relationship between fluorescent protein level (MMFI) and speed for individual frame–frame movements was displayed and analysed either by plotting the distribution of data or by statistical comparison of population of melanosomes containing different levels of associated fluorescent protein (see *Materials and Methods* for details).

To more rigorously examine the apparent differences in melanosomal Rab27a content and motility, we established a semi-automated method that allowed unbiased, quantitative analysis of the relationship between these parameters (see *Materials and Methods*; Figure 2). Briefly, movement of melanosomes and associated EGFP-tagged Rab/effector proteins was recorded using time-lapse confocal or TIRFM in thin, flat areas of cytoplasm in cells exhibiting a high ratio of membrane:cytosolic EGFP. Using Volocity image analysis software, pigmented melanosomes, visible in phase contrast images, were defined using size and intensity filters and then tracked through image sequences using the shortest path tracking model within the software. By this method, image sequences (100 frames) recorded from 20 × 20 µm regions of interest typically yielded 100–350 tracks that were greater than 10 frames long and could be visually authenticated. This corresponds to 2500–10 000 frame-to-frame melanosome movements from each time-lapse sequence.

To analyse the relationship between Rab/effector recruitment and melanosome speed, mean melanosome-associated fluorescence (MMFI) and frame-to-frame speed data were extracted for each tracked data point. This allowed direct comparison of these parameters; either using two-dimensional plots of the distribution of data and correlation testing (Figure 1E) or by ranking by MMFI and binning into 10 equal groups each containing 10% of the total data (Figure 1F). The latter approach compensated for differences in expression level and allowed statistical testing of differences in speed data associated with melanosomes containing different levels of Rab/effector proteins in several cells (see also *Materials and Methods*; Figure 2). Similar procedures were used to examine the relationship between melanosome size and Rab/effector content.

### Rab27a is enriched on slow moving and static melanosomes

Using this method, we obtained and analysed speed and EGFP-Rab27a MMFI data of pigmented melanosome in EGFP-Rab27a-rescued melan-ash cells. Analysis of the total data set revealed a skewed speed distribution (mean median speed = 0.035 µm/second, *n* = 14 820 events from four different cells; Figure 1D). Similar analysis in fixed melanocytes showed overlap with living cells (mean median speed = 0.013 µm/second, *n* = 26 805 events from four cells; Figure 1D). This suggested that a significant percentage of melanosomes in living melanocytes are likely to be static. On the basis that 100% of the melanosomes in the fixed sample were static, we established a threshold speed of 0.05 µm/second (corresponding to the maximum melanosome speed observed in fixed cells), below which we considered them to be static. On the basis of this assumption, we found that 63.0% of melanosomes in EGFP-Rab27a-rescued melan-ash cells were static/docked.

To examine the role of Rab27a (and downstream effector) recruitment in regulation of melanosome movement, we tested the relationship between melanosome speed and EGFP-Rab27a MMFI for each frame-to-frame movement event (as described in *Materials and Methods*). The distribution of data indicates that melanosomes with high EGFP-Rab27a MMFI were more likely to move more slowly than those with low EGFP-Rab27a MMFI (Spearman *r* = −0.3572 with 95% confidence interval −0.3852 to −0.3286, p < 0.001; Figure 1E). Comparison of melanosome speed differences between EGFP-Rab27a MMFI populations (0–10%, 10–20%, etc.) confirmed that melanosomes with lower EGFP-Rab27a MMFI (0–10%) group move significantly faster (mean median speed = 0.045 µm/second) than those with higher EGFP-Rab27a MMFI (i.e. all other sub-population, mean median speed = 0.030 µm/second; Figure 1F, squares). This difference reflects a lower proportion of static melanosomes in the 0–10% group (55.4%) compared with the other populations (e.g. 73.3% of the 90–100% population; Figure 1F, bars), and also a small but significant increase in the mean median speed among motile melanosomes (speed > 0.05 µm/second) in the 0–10% group compared with others (*Supporting Information* and Figure 1F, circles).

Similar results were obtained using wild-type melan-ink4a melanocytes expressing EGFP-Rab27a. Analysis of the complete data set (i.e. both motile and static movement events) revealed that the 0–10% and 10–20% groups were significantly faster than all other groups. As above, this was due to a lower proportion of static melanosomes in the 0–10% (65.8%) and 10–20% (71.6%) groups compared with other groups (20–100%, 78.5%), and a small but significant increase in speed of moving melanosomes in the 0–10% and 10–20% populations compared with higher EGFP-Rab27a MMFI groups (Figure S1A,B and *Supporting Information*). The higher overall level of static melanosomes in EGFP-Rab27a expressing melan-ink4a (76.5%) versus EGFP-Rab27a-rescued melan-ash (68.6%) is likely due to the presence of endogenous Rab27a in melan-ink4a.

Finally, similar results were observed for EGFP-Rab27b-rescued melan-ash and EGFP-Rab27b-expressing melan-ink4a cells (Figure S1C–F and *Supporting Information*).

These results suggest that recruitment of EGFP-Rab27a reduced the frequency and speed of melanosome movement. Due to the relatively short time over which time-lapse sequences were recorded (100–150 seconds), we only infrequently observed apparent Rab27/effector association/dissociation with individual melanosomes (Movie S1 and Figure 1C white arrows and Movie S11 arrows).

### Rab32/38 is enriched on fast-moving melanosomes that have small melanin cores

We next investigated the specificity of the relationship between Rab27 recruitment and reduced melanosome speed/frequency of movement. To do this, we investigated the correlation between membrane recruitment of other Rabs and melanosome movement, focusing on those whose function has been linked to melanosomes; namely Rab32, Rab38, Rab3a, Rab7 and Rab8. We observed little recruitment of either Rab7 or Rab8 to pigmented melanosomes in melan-ink4a cells precluding further investigation of their role in melanosome movement (data not shown) (32).

**Figure 3:**
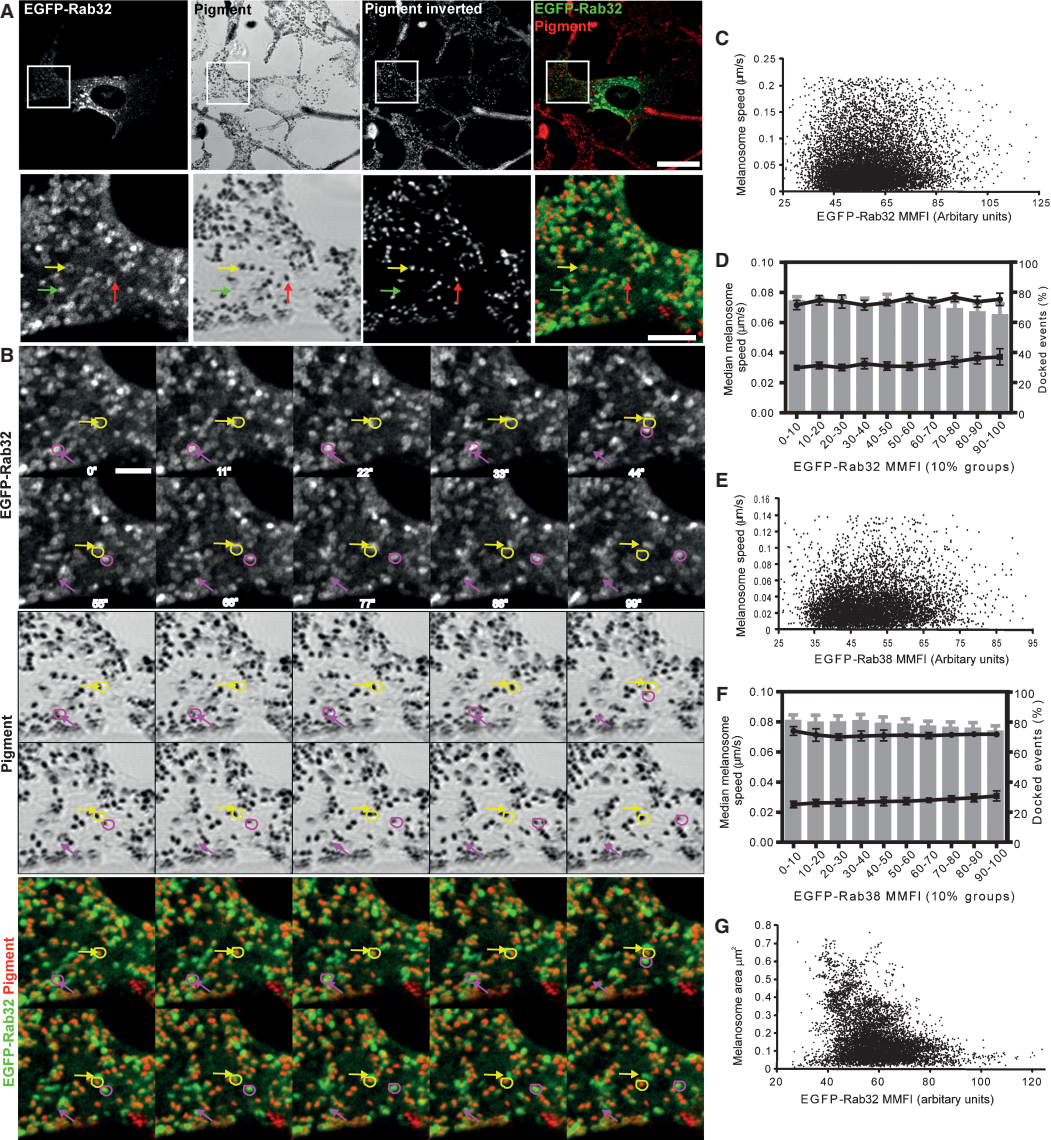
Rab32/38-positive melanosomes move more rapidly than do the Rab32/38-negative melanosomes The melan-ink4a melanocytes were transfected with a plasmid encoding EGFP-Rab32 or EGFP-Rab38 and imaged alive using a confocal microscope. A) The upper panels show an overview of the EGFP-Rab32 expressing melan-ink4a cell used for time-lapse recording. The white box indicates the area of cytoplasm used for time-lapse recording (B). The lower panels show a single frame from a time-lapse series (B, *t* = 0) revealing that cells contain EGFP-Rab32-positive melanosomes, EGFP-Rab32-negative melanosomes and EGFP-Rab32-positive vesicles lacking pigment (indicated by yellow, red and green arrows). White arrows indicate EGFP-Rab32-positive vesicles located close to pigmented melanosomes. In the overlay image, green and red correspond to EGFP-Rab32 and pigmented melanosomes. Scale bar = 20 µm for upper and 5 µm for lower panels. B) Images from a time-lapse series (numbers indicate elapsed time in seconds; Movie S3) showing the movement of pigmented melanosomes and EGFP-Rab32 (red and green, respectively, in colour combine). Yellow and magenta rings indicate the position of EGFP-Rab32-positive and Rab32-negative melanosomes in each frame, while corresponding coloured arrows show the starting position of each melanosome. Scale bar = 3 µm. C and E) Scatter plots showing the relationship between EGFP-Rab32 and EGFP-Rab38 MMFI and speed for each movement event in melan-ink cells expressing these proteins (*n* = 11 377 and 11 983 events recorded from one representative cell for each condition). D and F) Data were grouped on the basis of melanosome-associated EGFP-Rab32 (*n* = 53 270 events) (D) or EGFP-Rab38 (*n* = 51 860 events) (F) signal intensity (MMFI), and median speed (mean and SEM of data from four cells shown for each) was plotted for each group. Data within each MMFI bin was filtered (see text) and the median speed of motile events (mean and SEM, line plot circles) and the percentage of static events (mean and SEM, bar plot right-hand *y*-axis) are displayed. Significance testing was conducted as described in *Materials and Methods* and is present in *Supporting Information*. G) The relationship between EGFP-Rab32a MMFI and melanosome size for each event in a melan-ink4a cell expressing EGFP-Rab32 (Spearman *r* = −0.2893 with 95% confidence interval −0.3099 to −0.2684, p < 0.001, *n* = 7968 movement events from a time-lapse series of one representative cell).

**Figure 4:**
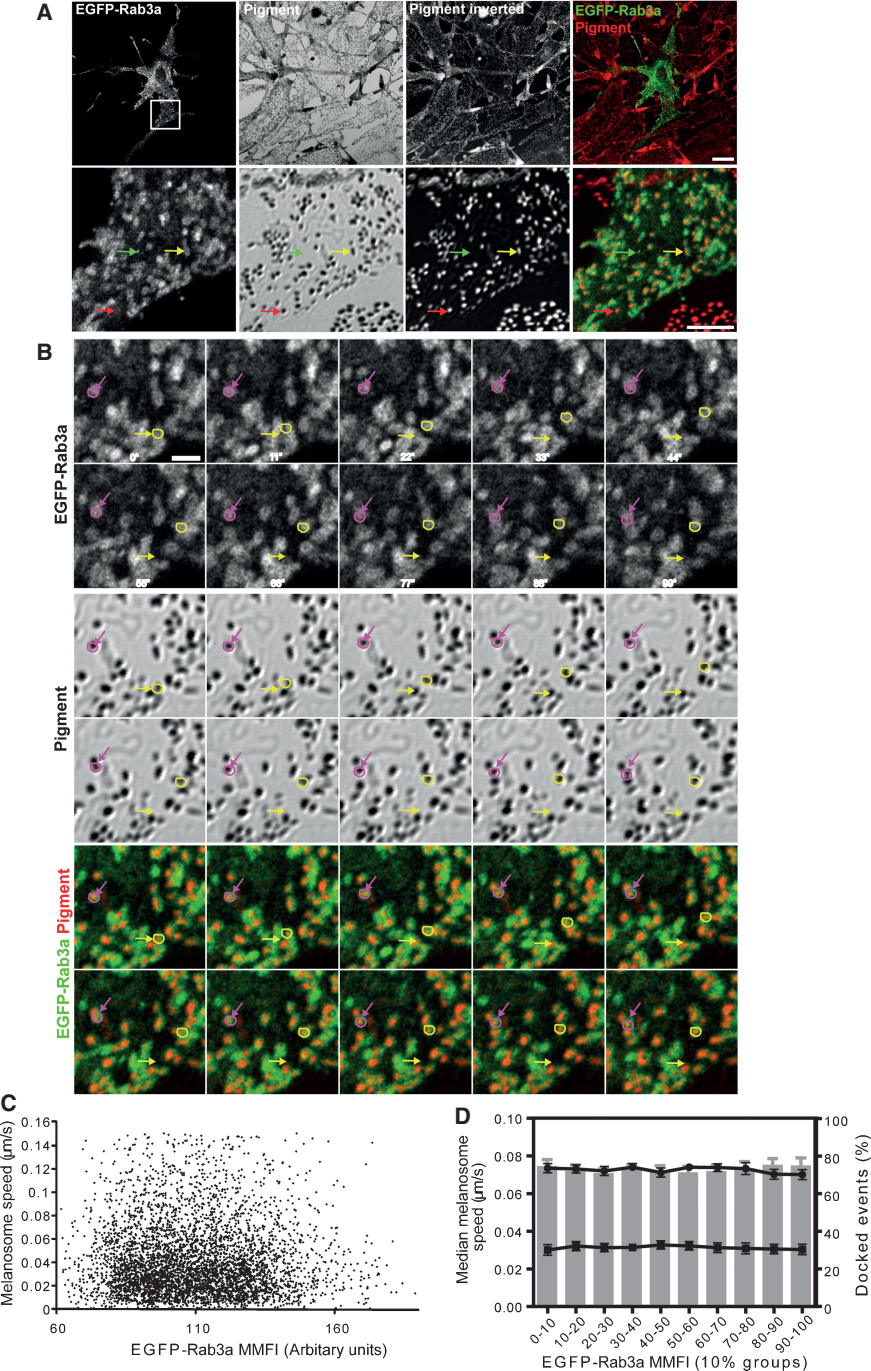
Rab3a-positive melanosomes move at the same speed as Rab3a-negative melanosomes The melan-ink4a melanocytes were transfected with a plasmid encoding EGFP-Rab3a and imaged alive using a confocal microscope. A) The upper panels show an overview of the EGFP-Rab3a expressing melan-ink4a cell used for time-lapse recording. The white box indicates the area of cytoplasm used for the time-lapse recording. The lower panels are a single frame from a time-lapse series showing that cells contain EGFP-Rab3a-positive melanosomes, EGFP-Rab3a-negative melanosomes and EGFP-Rab3a-positive vesicles lacking pigment (indicated by yellow, red and green arrows). In the overlay image, green and red correspond to EGFP-Rab3a and pigmented melanosomes. Scale bar = 20 µm for upper and 5 µm for lower panels. B) Images from a time-lapse series (numbers indicate elapsed time in seconds; Movie S5) showing the movement of pigmented melanosomes and EGFP-Rab27a (red and green, respectively, in colour combine). Yellow and magenta rings indicate the position of EGFP-Rab3a-positive and EGFP-Rab3a-negative melanosomes in each frame, while corresponding coloured arrows show the starting position of each melanosome. Scale bar = 2.5 µm. C) A scatter plot showing the relationship between EGFP-Rab3a MMFI and speed for each movement event in a melan-ink cell expressing EGFP-Rab3a (*n* = 4678 events recorded from one representative cell). D) Data were binned on the basis of melanosome-associated EGFP-Rab3a MMFI, and median speed (mean and SEM of data from four cells shown, a total of 54 010 movement events were analysed) was plotted for each group. Data within each MMFI bin was filtered (see text) and the median speed of motile events (mean and SEM, line plot circles) and the percentage of static events (mean and SEM, bar plot right-hand *y*-axis) are displayed. Significance testing was conducted as described in *Materials and Methods* and is present in *Supporting Information*.

As with Rab27a, confocal imaging of melan-ink4a cells expressing EGFP-Rab32/38 revealed three populations of cytoplasmic structures: (i) melanosomes containing EGFP-Rab (∼83%), (ii) melanosomes free of EGFP-Rab (∼7%) and (iii) EGFP-Rab-positive vesicles that appeared to lack a melanin core (10%; Figure 3A, 4A and S2A, yellow, red and green arrows). Observation of time-lapse image sequences of melan-ink4a cells expressing EGFP-Rab32/38 appeared to show that melanosomes containing lower MMFI move more slowly than those with higher MMFI (Figures 3B and S2B and Movies S3 and S4). Tracking and analysis revealed that melanosomes with higher EGFP-Rab32/38 MMFI (90–100% populations) indeed moved significantly faster than populations containing lower EGFP-Rab32/38 MMFI (0–10% to 60–70% groups, p < 0.05; Figure 3C–F and *Supporting Information*). This was consistent with the finding that the 90–100% population was enriched in undocked/fast-moving melanosomes relative to others (Figure 3C–F). For Rab32- and Rab38-expressing cells, 65.0 and 73.9% in the 90–100% group were static versus 74.2 and 81.0% in the corresponding 0–10% group. Interestingly, melanosomes with high EGFP-Rab32/38 MMFI have relatively small melanin cores (i.e. there is a negative correlation between size and MMFI; Spearman *r* = −0.2893 with 95% confidence interval −0.3099 to −0.2684, p < 0.001; Figures 3G and S2C). These observations are in line with the idea that Rab32/38 regulates melanogenesis and hence associates with a distinct population of pigmented melanosomes from Rab27 that may be less mature given their relatively lower frequency of docking and small melanin core size.

Recruitment of Rab27-related protein, Rab3a, to melanosomal membranes in melan-ink4a did not show any significant correspondence with differences in speed of movement (either for the total or motile events) or proportion of docked versus motile granules (Figure 4B–D, Movie S5 and *Supporting Information*). This indicates the specificity of the recruitment of Rab27a and Rab32/38 to slow- and fast-moving sub-populations of melanosomes. Interestingly, although both Rab3a and Rab27a fall within the same Rab functional group and share some effector interactions, the finding that Rab3a recruitment had little effect on melanosome motility is consistent with *in vitro* data showing that Rab3a does not interact with Mlph (22).

### Rab27a and Rab32/38 are present on distinct populations of melanosomes with differing movement characteristics

**Figure 5:**
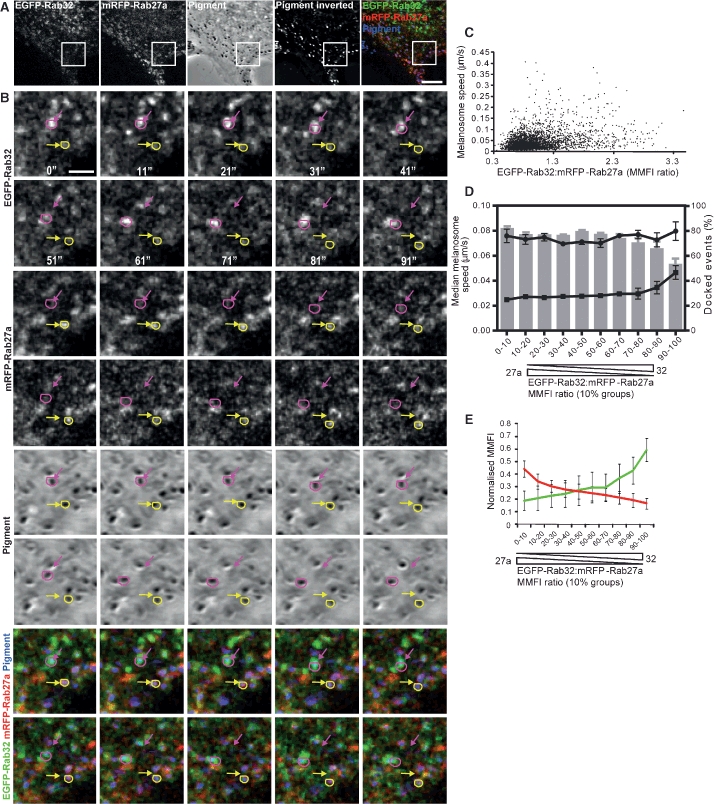
Rab27a and Rab32/38 are present on different populations of melanosomes that move at different speeds The melan-ink4a melanocytes were co-transfected with plasmids encoding EGFP-Rab32 and mRFP-Rab27a. A) The upper panels show an overview of a section of the peripheral cytoplasm of the EGFP-Rab32 and pmRFP-Rab27a expressing melan-ink4a cell used for time-lapse recording (B). The white box indicates the area of cytoplasm used for the time-lapse recording. B) Images from time-lapse series (numbers indicate elapsed time in seconds; Movie S6) showing the movement of pigmented melanosomes, EGFP-Rab32 and mRFP-Rab27a (blue, green and red, respectively, in the colour combine). Yellow and magenta rings indicate the position of mRFP-Rab27a-positive and EGFP-Rab32-positive melanosomes in each frame, while corresponding coloured arrows show the starting position of each melanosome. Scale bar = 8 µm for (A) and 3 µm for (B). C) A scatter plot showing the relationship between the ratio of melanosome-associated EGFP-Rab32:mRFP-Rab27a signal intensities and speed for each movement event in a melan-ink cell expressing these proteins (*n* = 3194 events recorded from one representative cell). D) Tracking data were grouped on the basis of the ratio of melanosome-associated EGFP-Rab32:mRFP-Rab27a signal intensities, and median speed (mean and SEM of data from three cells shown, a total of 7794 movement events were analysed) was plotted for each group. Data within each MMFI ratio bin was filtered (see text) and the median speed of motile events (mean and SEM, line plot circles) and the percentage of static events (mean and SEM, bar plot right-hand *y*-axis) are displayed. Significance testing was conducted as described in *Materials and Methods* and is present in *Supporting Information*. E) Mean normalized fluorescence intensity (±SD) for EGFP-Rab32 and mRFP-Rab27a within each EGFP-Rab32:mRFP-Rab27a group (D).

Our findings thus far indicated that Rab32/38 and Rab27 reside on distinct populations of melanosomes. Rab32/38 associated with small melanin-cored, fast-moving melanosomes and Rab27a associated with slow-moving/static melanosomes. To more directly examine the relationship between melanosome movement and Rab content (Rab27 versus Rab32/38), we recorded time-lapse image sequences of melanosome and fluorescent protein movement in melan-ink4a cells transiently co-expressing mRFP-Rab27a and EGFP-Rab32, tracked the melanosomes, and compared Rab content with movement. Visual inspection revealed that Rab27a-positive melansomes are more peripheral than Rab32-positive (and Rab38) melanosomes, consistent with previous observations (20) (Figure 5A and Movie S6). Additionally, we observed that fast movements are more frequent in melanosome populations containing higher Rab32:Rab27a MMFI ratio compared with other populations (the 90–100% group is significantly faster than all the other groups, p < 0.01; *Supporting Information and*Figure 5B–D). This difference in overall speed reflects the finding that all other groups (0–10% to 80–90%) are enriched in docked and slower motile melanosomes compared with the 90–100% population (75.5% versus 52.3%; *Supporting Information*). Analysis of the intensity of individual fluorophores underlying the changes in EGFP:mRFP ratio confirmed that the 0–10% group represents Rab27a-positive and Rab32-negative melanosomes, while 90–100% represents Rab27a-negative and Rab32-positive melanosomes (Figure 5E). Similar results were obtained for melanosome speed and EGFP-Rab38:mRFP-Rab27a MMFI ratio (data not shown). This confirms that Rab32/38 and Rab27a predominantly reside on distinct populations of fast- and slow-moving/static melanosomes.

### The correlation between Rab27a-status and melanosome speed is dependent upon effector Mlph

**Figure 6:**
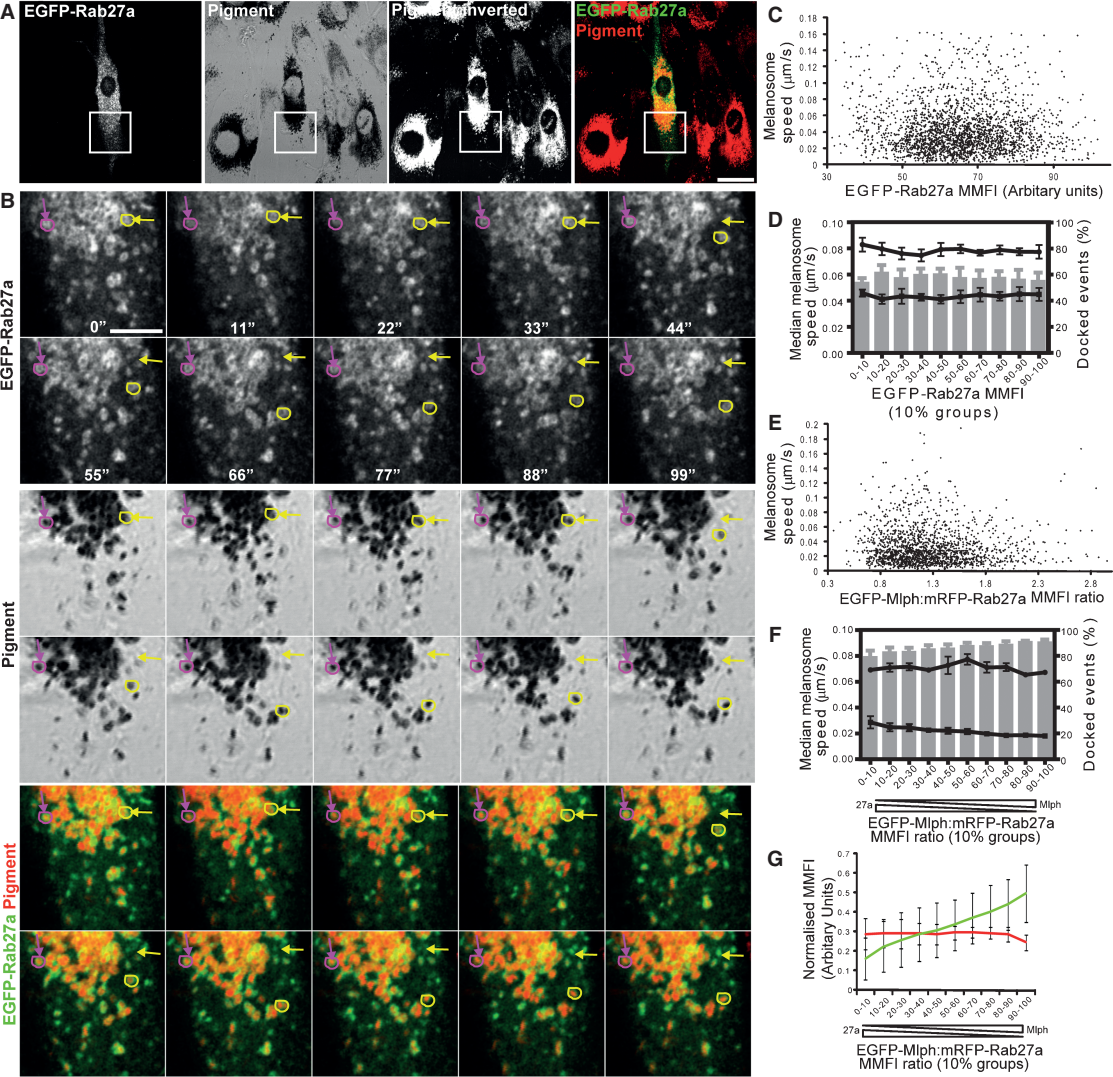
In the absence of effector Mlph, Rab27a-positive and Rab27a-negative melanosomes move at the same speed Mlph-null (melan-ln) melanocytes were transfected with a plasmid encoding EGFP-Rab27a, and imaged alive using a confocal microscope. A) The upper panels show an overview of the EGFP-Rab27a expressing melan-ln cell used for time-lapse recording. The white box indicates the area of cytoplasm used for the time-lapse recording. B) Images from a time-lapse series (numbers indicate elapsed time in seconds; Movie S7) showing the movement of pigmented melanosomes and EGFP-Rab27a (red and green, respectively, in the colour combine). Yellow and magenta circles indicate the position of EGFP-Rab27a-positive and EGFP-Rab27a-negative melanosomes in each frame, while corresponding coloured arrows show the starting position of each melanosome. Scale bars = 25 µm for (A) and 10 µm for (B). C and E) Scatter plots showing the relationship between EGFP-Rab27a MMFI and speed in melan-ln and the ratio of EGFP-Mlph:mRFP-Rab27a MMFI and speed in melan-ink4a, respectively (*n* = 2361 and 1857 events recorded from one representative cell for each condition). D and F) Tracking data (corresponding to C and E) were binned on the basis of melanosome-associated EGFP-Rab27a MMFI (D) or EGFP-Mlph:mRFP-Rab27a MMFI ratio (F), and median speed [mean and SEM of data from four cells (9623 events) (D) or three cells (10 568) events] was plotted for each group (line plot squares). Data within each MMFI bin was filtered (see text) and the median speed of motile events (mean and SEM, line plot circles) and the percentage of static events (mean and SEM, bar plot right-hand *y*-axis) are displayed. Significance testing was conducted as described in *Materials and Methods* and is present in *Supporting Information*. G) Mean median normalized fluorescence intensity (±SD) for EGFP-Mlph and mRFP-Rab27a within each EGFP-Mlph:mRFP-Rab27a group (F).

We next investigated the mechanism by which Rab27 recruitment correlated with reduced melanosome speed/movement frequency. Previous studies suggested that Rab27a sequentially recruits the modular adaptor Mlph and alternative myosin motor MyoVa, and that this complex allows actin-dependent capture of melanosomes in peripheral dendrites (23). Our observation that recruitment of EGFP-Rab3a, related to Rab27 but unable to interact with Mlph (22), showed no correlation with slow melanosome movement (Figure 5) indicated a requirement for interaction with Mlph in reduction of melanosome speed. To test this more directly in our live-cell tracking system, we examined the relationship between EGFP-Rab27a MMFI and speed in melan-ln melanocytes (immortal Mlph-null (33)). As expected, we observed that EGFP-Rab27a was unable to restore melanosome transport to peripheral dendrites in these cells (Figure 6A). As above (Figure 1), three classes of structure were observed: EGFP-Rab27a-positive melanosomes, EGFP-Rab27a-negative melanosomes and melanin-free EGFP-Rab27a-positive vesicles (Figure 6B and Movie S7). Tracking and analysis indicated that melanosomes in EGFP-Rab27a-expressing melan-ln cells more frequently undertake fast movements (median speed = 0.043 µm/second) than melanosomes in melan-ink4a and rescued melan-ash (median speed = 0.035 µm/second; Figure 1D). However, this may be due to the fact that it was only possible to track the small proportion of melanosomes that emerged from the perinuclear cluster of melanosomes, as the others (the majority) were in such close proximity as to preclude automated tracking of their movements. Nevertheless, comparison of melanosome speed (for total and motile events) and percentage docked with EGFP-Rab27a MMFI did not reveal any relationship between these parameters (Figure 6C,D). This suggests that the reduction in melanosome speed and increased docking observed on recruitment of EGFP-Rab27a in melan-ash and melan-ink4a is Mlph dependent.

Also consistent with this, in melan-ink4a cells co-expressing EGFP-Mlph and mRFP-Rab27a, we observed that higher Mlph:Rab27a MMFI ratio was associated with slower overall melanosome speed (the 0–10% group was significantly faster than the groups 30–40% to 90–100%, p < 0.001) due to the relative enrichment in static melanosomes in the high Mlph:Rab27a (86.9%) versus low Mlph:Rab27a population (74.5%; Figure 6E,F and *Supporting Information*). Analysis of the changes in the intensity of individual fluorophores underlying these changes in EGFP:mRFP indicates that this effect corresponds to the recruitment of Mlph onto Rab27a-positive melanosomes (Figure 6G).

### Rab27a recruitment switches melanosomes from MT- to actin-dependent transport

Finally, we used our melanosome-tracking system to evaluate the contribution of the MT and actin networks to the differences in speed/docking of melanosomes with different EGFP-Rab27a levels. For this, EGFP-Rab27a-rescued melan-ash melanocytes were treated with cytoskeleton-disrupting drugs, and imaged alive as before (Movies S8–S10). Nocodazole and cytochalasin D disrupt the MT- and F-actin networks, respectively.

**Figure 7:**
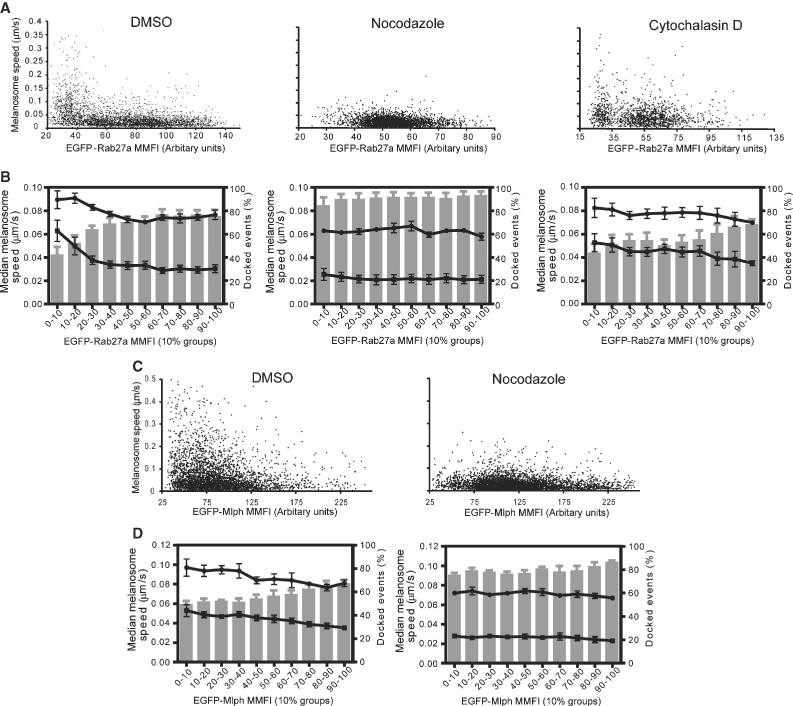
Recruitment of Rab27a and effectors to melanosomes correlates with switching of melanosomes from MT- to actin-dependent transport Rab27a-null (melan-ash, A, B) or Mlph-null (melan-ln, C, D) melanocytes were transfected with plasmids encoding EGFP-Rab27a (A and B) or EGFP-Mlph (C and D) and expressing rescuing levels of each transgene treated with the indicated cytoskeleton-disrupting drugs (or vehicle DMSO; see *Material and Methods*), and imaged alive 1 h later using a confocal microscope (A and B) or combined TIRFM/phase contrast microscopy (C and D). Scatter plots show the relationship between EGFP-Rab27a MMFI (A) or EGFP-Mlph MMFI (C) and speed in individual representative melan-ash or melan-ln cells, respectively. A) DMSO *n* = 3880, nocodazole *n* = 2821 and cytochalasin D *n* = 1512. C) DMSO *n* = 3537 and nocodazole *n* = 2522. B and D) Total tracking data (corresponding to A and C) were binned on the basis of melanosome-associated EGFP-Rab27a MMFI (A) or EGFP-Mlph:mRFP-Rab27a MMFI ratio (C), and median speed (mean and SEM of data from several cell in each condition) are displayed (line plot squares). Data within each MMFI bin was filtered (see text) and the median speed of motile events (mean and SEM, line plot circles) and the percentage of static events (mean and SEM, bar plot right-hand *y*-axis) are displayed. Significance testing was conducted as described in *Materials and Methods* and is present in *Supporting Information*. B) DMSO *n* = 14 820 events from four cells, 10 µm nocodazole *n* = 15 414 events from three cells and 10 µm cytochalasin D *n* = 8019 events from five cells. D) 0.1% DMSO *n* = 9366 events from four cells, 10 µm nocodazole and *n* = 17 045 events from four cells.

Tracking revealed that for dimethylsulphoxide (DMSO)-treated (vehicle) cells (as for untreated cells; Figure 1D) the lowest MMFI groups had significantly faster speeds (both total and motile events) and lower percentage of docked melanosomes than the others (p < 0.001, mean median 0.063 µm/second, 0–10% group and 0.050 µm/second, 10–20% group for totals; Figure 7A,B and *Supporting Information*). In contrast, we observed little difference in the speed distribution between the MMFI groups in nocodazole-treated cells. Indeed only the 0–10% group (total) was slightly faster than the other groups (p < 0.001, mean median = 0.025 µm/second, 0–10% group versus 0.023 µm/second, 10–20% group). This was most likely due to the slightly reduced percentage of static melanosomes in this group compared with others rather than differences in the speed of motile melanosome fraction (Figure 7B). Overall melanosome speed in nocodazole-treated cells was significantly (p < 0.001) reduced (median speed = 0.022 µm/second) compared with DMSO-treated (median speed = 0.035 µm/second) cells (Figures 1D and 7A,B and Movies S8 and S9). A large contribution to the global reduction in speed in nocodazole-treated cells was due to the increased proportion of static melanosomes in the cells (90.5%) compared with DMSO-treated cells (66.9%). Additionally, the overall speed of motile melanosomes in nocodazole-treated cells was lower than their DMSO-treated counterparts (nocodazole mean median speed = 0.062 µm/second and maximum speed = 0.214 µm/second versus 0.078 µm/second and 0.800 µm/second for DMSO; p < 0.001). Together these observations suggest that in DMSO-treated cells, the reduction in speed associated with EGFP-Rab27a recruitment was due to a reduction in the frequency of fast (probably MT-dependent) movement and an increase in slower Rab27a–Mlph–MyoVa-dependent movement/tethering. This is in agreement with the capture hypothesis, in which Rab27a and effector recruitment switches melanosomes from MT-dependent fast movement to F-actin-dependent slow moving/tethered state (9). Surprisingly, however, we did not observe an increase in speed (either total or motile) upon melanosomal recruitment of EGFP-Rab27a that might be expected if the Rab27a–Mlph–MyoVa complex itself transported melanosomes on F-actin (Figure 7A,B). Thus, our data are more consistent with a tethering role for MyoVa in melanosome transport. Similar results were obtained from studies of Mlph recruitment and melanosome movement in nocodazole-treated EGFP-Mlph-rescued melan-ln cells (Figure 7C,D and *Supporting Information*).

Next, to evaluate the contribution of the actin cytoskeleton to melanosome movements, we treated EGFP-Rab27a-rescued melan-ash cells with cytochalasin D. In contrast to nocodazole, this treatment increased overall melanosome speed (median speed = 0.043 µm/second, p < 0.001) compared with control cells (Figure 1D and Movie S10). This increase corresponded to the melanosome speed distribution observed in non-rescued Mlph-null cells expressing EGFP-Rab27a (Figures 1D and 6C,D), and is consistent with the release of a percentage of melanosomes from docking (55.6% in cytochalasin D versus 66.9% in DMSO-treated cells) and an increase in the frequency of faster (probably MT-dependent) movements. Comparison of speed with EGFP-Rab27a MMFI in these cells indicated an inverse relationship between these parameters, with faster movements more frequent in populations containing lower EGFP-Rab27a MMFI (the 0–10% group is faster than 60–70% to 90–100% groups, p < 0.01, while the 90–100% group is slower than all but the 80–90% group, p < 0.01; Figure 7A,B) and a reciprocal reduction in docking, which to some extent resembled the situation in untreated/DMSO-treated cells. However, the increase in melanosome speed among most EGFP-Rab27a MMFI populations (except the 0–10% and 10–20% groups) in cytochalasin D-treated cells indicated that recruitment of Rab27a–Mlph–MyoVa complex was less efficient in reducing melanosome speed in this condition (Figure 7A,B). This is consistent with the idea that Rab27a–Mlph–MyoVa-dependent reduction in speed is dependent upon the integrity of F-actin and that slower movements, more frequent in high EGFP-Rab27a and EGFP-Mlph MMFI groups, are likely to be dependent upon F-actin integrity. Nevertheless, the finding that there is some effect of Rab27a recruitment in reducing melanosome speed (and increasing melanosome docking) suggested that depolymerization of actin by cytochalasin D was incomplete in these cells. Interestingly, immunostaining of fixed cytochalasin D-treated melanocytes revealed that melanosomes retained Rab27a-positive F-actin punctae, suggesting that melanosomal Rab27a–Mlph–MyoVa complex might stabilize local F-actin (Figure S3). Additionally, this and other images of cytochalasin D-treated cells indicate that this treatment results in bundling of cytoplasmic MTs compared with untreated cells. Such bundling might reduce the efficiency of MT-dependent transport and may provide an explanation for the reduction in speed of the 0–10% and 10–20% populations in cytochalasin D compared with DMSO-treated cells.

Finally, comparison between melanosome speed in living cells in the presence or absence of cytoskeleton inhibitor and speed events in fixed cells indicated that MT- and F-actin-dependent movements range between 0.1–0.8 and 0.05–0.10 µm/second, respectively, and are relatively infrequent (∼10.5 and ∼26.5% of total events).

## Discussion

Here we established a new method that combines time-lapse confocal microscopy, to record the movement of melanosomes and associated proteins, with automated melanosome tracking that allowed large-scale statistical analysis of organelle movement parameters with peripheral membrane protein recruitment. This approach allowed us to more directly examine the role of such proteins in organelle transport, than was possible using previous manual tracking methods. We did this by using melanosomes and Rab/effector proteins in melanocytes as a model. This revealed several significant insights into these processes.

First, we found that Rab27a and Rab32/8 exist on distinct populations of melanosomes that differ in their intracellular distribution, size of their melanin core and movement speed. Specifically, we found that Rab27a-positive melanosomes move significantly more slowly than do the Rab32/38-positive melanosomes. On the basis of the results of cytoskeleton inhibitor treatments (Figure 7) and double EGFP-Rab32/38 and mRFP-Rab27a co-expression (Figure 5), it appears that Rab32/38-positive (Rab27a-negative) melanosomes more frequently undertake episodes of MT-dependent fast movement (0.1–0.8 µm/second) than Rab27a-positive (Rab32/38-negative) melanosomes. These observations are consistent with the idea that Rab32/38 regulate melanogenesis and are hence more likely to be present on less mature melanosome that move on MTs, in order to travel from the perinuclear cytoplasm into peripheral dendrites (20,32). In addition, these results suggest that a co-ordinated switching or conversion event triggers loss of Rab32/38 and its replacement with Rab27a may thereby integrate the processes of melanosome maturation with docking of mature melanosome in dendrites. Interestingly, other LRO-containing cell types, e.g. mast cells, platelets and alveolar epithelium cells type II (AETII), also express Rab32/38 and Rab27a, suggesting that similar organellar signalling networks may operate in these cell types (13). Identification of regulators and effectors of these Rabs may throw light on the mechanism of such Rab conversion, as has been reported in the mammalian endocytic pathway and secretory pathway in yeast (15).

Second, comparison of the speed of Rab27a-positive and Rab27a-negative melanosomes indicates that recruitment of Rab27a results in Mlph-dependent (and MyoVa-dependent) switching of melanosomes from MT- to actin-based transport. This is consistent with the conclusion of previous studies of MyoVa function in melanosome transport (9), as well as subsequent findings, that Rab27a and Mlph anchor and activate MyoVa on the melanosome membrane (23). However, the methodology used here has several advantages over that used previously, and allowed us to draw more direct conclusions on the role of the Rab27a–Mlph–MyoVa complex in melanosome transport. Importantly, by recording the movement of both melanosomes and associated proteins in cells containing dispersed melanosomes (melan-ink4a and rescued melan-ash and melan-ln), we were able to compare movement with Rab27 complex recruitment using organelles within the same cell, and thus draw direct conclusions on the relationship of these parameters. This is in contrast to previous studies that reached conclusions on MyoVa and MyoVIIa function based on differences in melanosome distribution and dynamics in myosin-null versus control melanocytes (9,28). Notably, in the study of MyoVa function, MyoVa-null melanocytes manifest perinuclear melanosome clustering, rendering the vast majority of melanosomes in these cells untrackable. Thus, comparison of movement between the control and MyoVa-null melanocytes is not straightforward.

Finally, we found that overall the speed of melanosome movement in melanocytes was considerably slower than previously reported [e.g. mean median = 0.035 µm/second (total events) and 0.078 µm/second (motile events) for EGFP-Rab27a-rescued melan-ash cells (this study; Figure 1D) versus previously published ∼0.6 µm/second average melanosome speed in control melanocytes] (9). One possible explanation for this is that automatic tracking was unable to follow fast-moving melanosomes through the crowded cytoplasm. However, visual confirmation of recorded tracks with observed movements indicated that the majority of movements were tracked regardless of speed. Indeed, the maximum melanosome speed recorded in many experiments ranged from 0.4 to 0.8 µm/second; however, these events were of low frequency. A second possibility is that melanosome movement in immortal melanocytes, such as melan-ink4a and melan-ash, is slower than in primary melanocytes used in previous studies. However, in our hands, automatic tracking analysis using primary melanocytes yielded similar results in terms of melanosome speed (data not shown). We consider the most likely explanation for these differences relates to the fact that automatic tracking allowed us to record a higher proportion of the total number of melanosome movements over the time course of the image sequences that was not feasible using manual tracking (9,28,29). Movies recording melanosome movement shown in this and previous studies indicate that the majority of melanosomes are slow moving or docked in living melanocytes. Consistent with this, our recordings are dominated by data derived from slow-moving/docked melanosomes and this strongly reduces our overall measurement of melanosome speed (median speed used throughout) compared with previous measurements (9,28,29). Nevertheless, we observed significant and reproducible differences in melanosome speed/docking frequency between groups of organelles positive and negative for Rab27a, effector Mlph and Rab32/38.

We conclude that automated correlative analysis of organelle movement and peripheral membrane protein distribution is likely to be a powerful approach for use in future studies to address the role of these proteins in organelle transport.

## Materials and Methods

### Plasmid expression vectors

The production of plasmid vectors allowing transient expression of EGFP-tagged Rab proteins pEGFP-Rab27a, pEGFP-Rab27b, pEGFP-Rab32, pEGFP-Rab38 and pEGFP-Mlph was described previously (20,32,33). pEGFP-Rab3a, allowing expression of human Rab3a fused to the C-terminus of EGFP, was produced by sub-cloning Rab3a coding sequence into pEGFPC2, between the *Eco*R1 and *Bam*H1 sites. pmRFP-Rab27a, allowing expression of rat Rab27a fused at the C-terminus of the monomeric red fluorescent protein, was produced by sub-cloning Rab27a coding sequence into pmRFP-C2, between the *Hin*dIII and *Bam*H1 restriction sites.

### Cell culture, transfection and cytoskeleton inhibitor treatment

Immortal mouse melanocytes melan-ink4a (wild-type derived from C57BL/6 mouse), melan-ash (Rab27a-null derived from the *Rab27a*^*ash/ash*^ mouse) and melan-ln (Mlph-null derived from the *Mlph*^*ln/ln*^ mouse) cell lines were maintained at 37°C, 10% CO_2_ in RPMI 1640 supplemented with 10% FBS, 100 U/mL penicillin G and 100 U/mL streptomycin, 200 nmol phorbol 12-myristate 13-acetate (PMA) and 200 pmol cholera toxin (32,33). Cells for transfection were seeded into glass-bottomed 35 mm dishes (Willco Wells) at 5 × 10^4^ cells/dish. Transient transfection was performed 24-h post-seeding using complexes comprised of 10 µL of Fugene6 (Roche), 500 µL serum-free OptiMEM (Invitrogen) and 2.5 µg of plasmid DNA per 35 mm dish. Complexes were incubated with cells for 4 h and then replaced with melanocyte growth medium (see above). After 24 h, melanocyte growth medium was replaced by L15 medium (Sigma) supplemented with 10% FBS, 100 U/mL penicillin G and 100 U/mL streptomycin and culture vessels were transferred to the pre-warmed (37°C) environmental chamber of a microscope for live-cell imaging. For drug treatments, cells were incubated in L15 medium supplemented with 10 µm nocodazole and/or 10 µm cytochalasin D (Sigma) from 10 mm stock dissolved in DMSO for 1 h at 37°C prior to imaging (in the presence of drug). Control cells were incubated for 1 h prior to imaging in medium supplemented with 0.1% DMSO.

### Live-cell imaging and automated tracking analysis

For live-cell confocal imaging, transfected cells were imaged using a Zeiss LSM-510 inverted confocal microscope (Carl Zeiss UK) using a 100× 1.4 NA Planapochromat oil-immersion lens. For two-colour recording (EGFP and transmitted light) the samples were scanned with the 488 nm line of an Argon laser, while for three-colour (EGFP, mRFP and transmitted light) samples were sequentially scanned (line mode) using the 488 nm line of an Argon laser and the 543 nm line of an HeNe laser. EGFP and mRFP signals were collected via 510–550 nm and 590–650 nm band pass filters. The confocal pinhole was set to 1 Airy Unit throughout and confocal laser power and detector gain were set to give optimal signal:noise ratio within the linear range of the detectors. All images presented are single *z*-plane sections. Two-colour image sequences (100 frames) were recorded at 1 frame/second (fps) and three-colour image sequences (50 frames) were acquired at 0.5 fps. For TIRFM imaging, a Zeiss Axiovert 200 inverted microscope modified for objective-type TIRFM (Till Photonics) was used. An argon laser 488 nm line was used to excite EGFP fluorescence through a 100× 1.4 NA phase contrast Zeiss Planapochromat objective. During observation, the cells were maintained on an MS2000 stage (Applied Scientific Instruments) equipped with a Focht chamber system (FCS2; Bioptechs) at 37°C. The images were recorded by a PCO SensiCam CCD camera at a rate of 1 fps. A Uniblitz shutter VS25 (Vincent Associates) in the transmitted light path was used to allow sequential acquisition of phase contrast images of melanosomes and TIRF images of EGFP. The scale was 16 pixels per micrometer.

### Data processing and analysis

To facilitate melanosome tracking, thin flat areas of melanocyte cytoplasm were selected as regions of interest in cells which exhibited high signal:noise ratio (melanosome-associated:cytosolic fluorescent protein). Image sequences were then imported to Volocity 5 image analysis software (Improvision) that allows automatic particle tracking. Melanosomes visible in transmitted light images were defined using intensity and size/area filters and then tracked using the shortest path tracking model within Volocity 5. Tracks shorter than 10 frames were removed, and the authenticity of remaining tracks was confirmed by visual inspection. Each image sequence typically yielded ∼100–350 tracks corresponding to ∼2500–10 000 frame-to-frame data points. To investigate the effects of Rab recruitment on organelle movement speed, the mean fluorescent intensity (MFI) associated with the melanosomes and frame-to-frame speed parameter were then extracted for each data point. To compensate for potential differences in expression level between cells, data points from each image sequence were ordered by MFI and binned into 10 equal groups each containing 10% of the data points (i.e. ∼250–1000 per group).

Bins from several cells were combined and tested for significant differences between groups using the Kruskal–Wallis test with Dunns post-test. For clarity, rather than these combined data sets, the figures show the mean (and standard error of the mean, SEM) of the median of each bin from several cells. For pairwise comparisons between full data sets, the Mann–Whitney test was performed. To assess possible correlations in scattered full data sets, Spearman rank correlation was performed. The significance interval 95% was used for all testing.

GraphPad Prism (GraphPad) was used for all graphing and statistical analysis. See online *Supporting Information* text for full details of statistical analysis of data. Images were processed for presentation using Photoshop CS software (Adobe) and movies were assembled using ImageJ software (NIH). For image sequences recorded from cells simultaneously expressing two fluorescent proteins, melanosome-associated MFI data was extracted for both fluorescent channels together with speed data. To examine the relationship between the levels of both proteins and melanosome speed, the ratio of EGFP MFI:mRFP MFI for each data point was calculated and then binned in 10% groups and analysed as above.

## Supporting Information

**Table S1: EGFP-Rab27a rescued melan-ash.**

**Table S2: melan-ink4a expressing EGFP-Rab32.**

**Table S3: melan-ink4a expressing EGFP-Rab3a.**

**Table S4: melan-ink4a expressing EGFP-Rab32 andmRFP-Rab27a.**

**Table S5: melan-ln expressing EGFP-Rab27a.**

**Table S6: EGFP-Rab27a rescued melan-ash.**

**Figure S1: Rab27b-positive melanosomes move more slowly thando the Rab27b-negative melanosomes.** The melan-ink4A (A, B, Eand F) and melan-ash (C and D) melanocytes were transfected withplasmids encoding EGFP-Rab27a (A and B) or EGFP-Rab27b (C–F),and the movement of melanosomes and associated EGFP-protein wererecorded and tracked as described in *Materials and Methods*. A, C and E) Scatter plots show the relationship between MMFI (forthe indicated Rab27 isoform) and speed in individual representativecells [for the indicated cell type and EGFP fusion; A, *n* =2496; C, *n* = 3569 and E, *n* = 6893 events]. B, D andF) Tracking data for several cells (corresponding to A, C and E)were binned on the basis of melanosome-associated EGFP-Rab27 MMFI,and the median speed (mean and SEM of data from four cells in eachcondition (B, *n* = 29 980; D, *n* = 24 817 and F,*n* = 24,179 events) was plotted for each group (line plotsquares). Data within each MMFI bin was filtered (see text) and themedian speed of motile events (mean and SEM, line plot circles) andthe percentage of static events (mean and SEM, bar plot right-hand *y*-axis) are displayed. Significance testing was conducted asdescribed in *Materials and Methods* and is present in *Supporting Information*.

**Figure S2: Rab38-positive melanosomes move more rapidly thando the Rab38-negative melanosomes.** The melan-ink4a melanocyteswere transfected with a plasmid encoding EGFP-Rab38 and imagedalive using a confocal microscope. A) The upper panels show anoverview of the EGFP-Rab38 expressing melan-ink4a cell used fortime-lapse recording. The white box indicates the area of cytoplasmused for time-lapse recording (B). The lower panels show a singleframe from a time-lapse series (B, *t* = 0) revealing thatcells contain EGFP-Rab38-positive melanosomes, EGFP-Rab38-negativemelanosomes and EGFP-Rab38-positive vesicles lacking pigment(indicated by yellow, red and green arrows). In the overlay image,EGFP-Rab38 = green and pigmented melanosomes = red. Scale bar = 20µm for upper and 5 µm for lower panels. B) Images froma time-lapse series (numbers indicate elapsed time in seconds;Movie S4) showing the movement of pigmented melanosomes andEGFP-Rab38 (red and green, respectively, in colour combine). Yellowand magenta rings indicate the position of EGFP-Rab38-positive andEGFP-Rab38-negative melanosomes in each frame, while correspondingcoloured arrows show the starting position of each melanosome.Scale bar = 5 µm. C) Scatter plot showing the relationshipbetween EGFP-Rab38 MMFI and melanosome size (*n* = 7039 movement events from time-lapse recording of one representative cell).

**Figure S3: The Rab27a–Mlph–MyoVa complex maystabilize melanosome-associated F-actin in melanocytes.** Themelan-c melanocytes were treated with 10 µM cytochalasin D orvehicle (0.1% DMSO) for 1 h and then fixed and stained with primaryantibodies specific for endogenous Rab27a and α-tubulin.Primary antibodies were detected using alexa-488- andalexa-647-labelled secondary antibodies, and F-actin distributionwas revealed using Texas red-conjugated phalloidin. Images werethen obtained using a confocal microscope (see *Materials andMethods*). Boxed area corresponds to digital zoom highmagnification bottom left inset, Scale bar = 20 µm.

Unless otherwise stated, all movies are assembled from 100 images acquired at a rate of ∼1 Hz using a confocal microscope(see *Materials and Methods*). Playback of all movies is 10× real-time.

**Movie S1:** Dynamics of EGFP-Rab27a containing structures (green) and melanosomes (pseudo-coloured red) in Rab27a-null (melan-ash) melanocytes expressing rescuing levels of EGFP-Rab27a. Magenta and yellow arrows highlight differences in the movement of individual EGFP-positive and EGFP-negative melanosomes. White arrows indicate a melanosome that appears to dissociate from a cluster of EGFP-Rab27a structures.

**Movie S2:** Rab27a-null (melan-ash) melanocytes expressingrescuing levels of EGFP-Rab27a were imaged using a TIRFM and phasecontrast modes (see *Materials and Methods*). The movie showsthe movement of EGFP-Rab27a containing structures (green) andmelanosomes (pseudo-coloured red). White arrows and arrowheadshighlight the movement of Rab27a-positive melanin-free tubules andvesicles (*n* = 60 frames).

**Movie S3:** Dynamics of EGFP-Rab32 containing structures (green) and melanosomes (pseudo-coloured red) in transfected melan-ink4a cells. Magenta and yellow arrows highlight differences in the movement of individual EGFP-positive and EGFP-negative melanosomes.

**Movie S4:** Dynamics of EGFP-Rab38 containing structures (green) and melanosomes (pseudo-coloured red) in transfected melan-ink4a cells. Magenta and yellow arrows highlight differences in the movement of individual EGFP-positive and EGFP-negative melanosomes.

**Movie S5:** Dynamics of EGFP-Rab3a containing structures (green) and melanosomes (pseudo-coloured red) in transfected melan-ink4a cells. Magenta and yellow arrows highlight differences in the movement of individual EGFP-positive melanosomes.

**Movie S6:** Dynamics of EGFP-Rab32 (green) and mRFP-Rab27a(red) containing structures and melanosomes (pseudo-coloured blue)in transfected melan-ink4a cells. Magenta and yellow arrows inevery fifth (*n* = 50 frames, 0.5 Hz acquisition rate) frame highlight the movement of individual EGFP-Rab32-positive and mRFP-Rab27a-positive melanosomes.

**Movie S7:** Dynamics of EGFP-Rab27a containing structures (green) and melanosomes (pseudo-coloured red) in Mlph-null (melan-ln) melanocytes expressing EGFP-Rab27a. Magenta and yellow arrows highlight differences in the movement of individual EGFP-positive melanosomes.

**Movie S8:** Dynamics of EGFP-Rab27a containing structures(green) and melanosomes (pseudo-coloured red) inEGFP-Rab27a-rescued Rab27a-null (melan-ash) melanocytes expressinglevels and incubated with DMSO (0.1%; vehicle control) for 60 minat 37°C.

**Movie S9:** Dynamics of EGFP-Rab27a containing structures(green) and melanosomes (pseudo-coloured red) inEGFP-Rab27a-rescued Rab27a-null (melan-ash) melanocytes expressinglevels and incubated with 10 µM nocodazole for 60 min at37°C, to disrupt the integrity of the MT cytoskeleton, prior toimaging.

**Movie S10:** Dynamics of EGFP-Rab27a containing structures(green) and melanosomes (pseudo-coloured red) in EGFP-Rab27arescued Rab27a-null (melan-ash) melanocytes expressing levels andincubated with 10 µM cytochalasin D for 60 min at 37°C,to disrupt the integrity of the actin cytoskeleton, prior toimaging.

**Movie S11:** Dynamics of EGFP-Mlph containing structures(green) and melanosomes (pseudo-coloured red) in EGFP-Mlph-rescuedMlph-null (melan-ln) melanocytes imaged using TIRFM and phasecontrast modes (see *Materials and Methods*). Magenta andyellow arrows (*n* = 20 frames) highlight melanosomes that appear to associate with and dissociate from peripheral clusters of EGFP-Mlph.
