# Supplementary material for: Semi-Automated Analysis of Organelle Movement and Membrane Content: Understanding Rab-Motor Complex Transport Function
Source: Traffic. 2011 Oct 11;12(12):1686–701. doi: 10.1111/j.1600-0854.2011.01283.x (PMC3264752; doi:10.1111/j.1600-0854.2011.01283.x)
Supplement: Supplementary file 1 [file tra0012-1686-SD1.doc]

**Supporting information.**

**Table S1. EGFP-Rab27a rescued melan-ash.**

Analysis of all speed events.

| Kruskal-Wallis test |  |  |  |
| --- | --- | --- | --- |
| P value | P<0.0001 |  |  |
| Exact or approximate P value? | Gaussian Approximation |  |  |
| P value summary | *** |  |  |
| Do the medians vary signif. (P < 0.05) | Yes |  |  |
| Number of groups | 10 |  |  |
| Kruskal-Wallis statistic | 192.0 |  |  |
|  |  |  |  |
| Dunn's Multiple Comparison Test | Difference in rank sum | Significant? P < 0.05? | Summary |
| 0-10 green vs 10-20% | 1075 | Yes | *** |
| 0-10 green vs 20-30 | 1204 | Yes | *** |
| 0-10 green vs 30-40 | 1701 | Yes | *** |
| 0-10 green vs 40-50 | 1424 | Yes | *** |
| 0-10 green vs 50-60 | 1395 | Yes | *** |
| 0-10 green vs 60-70 | 1187 | Yes | *** |
| 0-10 green vs 70-80 | 1084 | Yes | *** |
| 0-10 green vs 80-90 | 1340 | Yes | *** |
| 0-10 green vs 90-100 | 1566 | Yes | *** |
| 10-20% vs 30-40 | 625.9 | Yes | *** |
| 10-20% vs 90-100 | 491.2 | Yes | * |
| 20-30 vs 30-40 | 497.2 | Yes | * |
| 30-40 vs 60-70 | -514.4 | Yes | * |
| 30-40 vs 70-80 | -617.5 | Yes | *** |
| 70-80 vs 90-100 | 482.8 | Yes | * |

**Analysis of motile events (speed >0.05**m/s)

| Kruskal-Wallis test |  |  |  |
| --- | --- | --- | --- |
| P value | < 0.0001 |  |  |
| Exact or approximate P value? | Gaussian Approximation |  |  |
| P value summary | **** |  |  |
| Do the medians vary signif. (P < 0.05) | Yes |  |  |
| Number of groups | 10 |  |  |
| Kruskal-Wallis statistic | 35.99 |  |  |
|  |  |  |  |
| Dunn's Multiple Comparison Test | Difference in rank sum | Significant? P < 0.05? | Summary |
| 0-10 vs 20-30 | 246.1 | Yes | * |
| 0-10 vs 30-40 | 339.9 | Yes | *** |
| 0-10 vs 40-50 | 363.0 | Yes | *** |
| 0-10 vs 60-70 | 259.0 | Yes | * |

**Analysis of docked events (%) (speed <0.05**m/s)

| One-way analysis of variance |  |  |  |
| --- | --- | --- | --- |
| P value | 0.5252 |  |  |
| P value summary | ns |  |  |
| Are means signif. different? (P < 0.05) | No |  |  |

**Table S2. melan-ink4a expressing EGFP-Rab32.**

Analysis of all speed events.

| Kruskal-Wallis test |  |  |  |
| --- | --- | --- | --- |
| P value | P<0.0001 |  |  |
| Exact or approximate P value? | Gaussian Approximation |  |  |
| P value summary | *** |  |  |
| Do the medians vary signif. (P < 0.05) | Yes |  |  |
| Number of groups | 10 |  |  |
| Kruskal-Wallis statistic | 192.0 |  |  |
|  |  |  |  |
| Dunn's Multiple Comparison Test | Difference in rank sum | Significant? P < 0.05? | Summary |
| 0-10 green vs 30-40 | -811.4 | Yes | * |
| 0-10 green vs 60-70 | -825.1 | Yes | * |
| 0-10 green vs 70-80 | -1463 | Yes | *** |
| 0-10 green vs 80-90 | -1994 | Yes | *** |
| 0-10 green vs 90-100 | -2293 | Yes | *** |
| 10-20% vs 70-80 | -1038 | Yes | *** |
| 10-20% vs 80-90 | -1570 | Yes | *** |
| 10-20% vs 90-100 | -1868 | Yes | *** |
| 20-30 vs 70-80 | -1194 | Yes | *** |
| 20-30 vs 80-90 | -1726 | Yes | *** |
| 20-30 vs 90-100 | -2025 | Yes | *** |
| 30-40 vs 80-90 | -1183 | Yes | *** |
| 30-40 vs 90-100 | -1482 | Yes | *** |
| 40-50 vs 70-80 | -976.4 | Yes | ** |
| 40-50 vs 80-90 | -1508 | Yes | *** |
| 40-50 vs 90-100 | -1807 | Yes | *** |
| 50-60 vs 70-80 | -955.6 | Yes | ** |
| 50-60 vs 80-90 | -1487 | Yes | *** |
| 50-60 vs 90-100 | -1786 | Yes | *** |
| 60-70 vs 80-90 | -1169 | Yes | *** |
| 60-70 vs 90-100 | -1468 | Yes | *** |
| 70-80 vs 90-100 | -830.3 | Yes | * |

**Analysis of motile events (speed >0.05**m/s)

| Kruskal-Wallis test |  |  |  |
| --- | --- | --- | --- |
| P value | 0.0029 |  |  |
| Exact or approximate P value? | Gaussian Approximation |  |  |
| P value summary | ** |  |  |
| Do the medians vary signif. (P < 0.05) | Yes |  |  |
| Number of groups | 10 |  |  |
| Kruskal-Wallis statistic | 25.07 |  |  |
|  |  |  |  |
| Dunn's Multiple Comparison Test | Difference in rank sum | Significant? P < 0.05? | Summary |
| 30-40 vs 90-100 | -467.1 | Yes | ** |
| 40-50 vs 90-100 | -427.7 | Yes | * |

**Analysis of docked events (%) (speed <0.05**m/s)

| One-way analysis of variance |  |
| --- | --- |
| P value | 0.8923 |
| P value summary | ns |
| Are means signif. different? (P < 0.05) | No |

**melan-ink4a expressing EGFP-Rab38.**

Analysis of all speed events.

| Kruskal-Wallis test |  |  |  |
| --- | --- | --- | --- |
| P value | < 0.0001 |  |  |
| Exact or approximate P value? | Gaussian Approximation |  |  |
| P value summary | **** |  |  |
| Do the medians vary signif. (P < 0.05) | Yes |  |  |
| Number of groups | 10 |  |  |
| Kruskal-Wallis statistic | 146.0 |  |  |
|  |  |  |  |
| Dunn's Multiple Comparison Test | Difference in rank sum | Significant? P < 0.05? | Summary |
| 0-10 vs 60-70 | -1128 | Yes | *** |
| 0-10 vs 70-80 | -1430 | Yes | *** |
| 0-10 vs 80-90 | -1767 | Yes | *** |
| 0-10 vs 90-100 | -2162 | Yes | *** |
| 10-20 vs 70-80 | -1051 | Yes | *** |
| 10-20 vs 80-90 | -1387 | Yes | *** |
| 10-20 vs 90-100 | -1782 | Yes | *** |
| 20-30 vs 70-80 | -940.1 | Yes | ** |
| 20-30 vs 80-90 | -1276 | Yes | *** |
| 20-30 vs 90-100 | -1672 | Yes | *** |
| 30-40 vs 70-80 | -885.1 | Yes | * |
| 30-40 vs 80-90 | -1221 | Yes | *** |
| 30-40 vs 90-100 | -1617 | Yes | *** |
| 40-50 vs 70-80 | -893.0 | Yes | ** |
| 40-50 vs 80-90 | -1229 | Yes | *** |
| 40-50 vs 90-100 | -1625 | Yes | *** |
| 50-60 vs 80-90 | -1079 | Yes | *** |
| 50-60 vs 90-100 | -1474 | Yes | *** |
| 60-70 vs 90-100 | -1035 | Yes | *** |

**Analysis of motile events (speed >0.05**m/s)

| Kruskal-Wallis test |  |
| --- | --- |
| P value | 0.3017 |
| Exact or approximate P value? | Gaussian Approximation |
| P value summary | ns |
| Do the medians vary signif. (P < 0.05) | No |
| Number of groups | 10 |
| Kruskal-Wallis statistic | 10.63 |

**Analysis of docked events (%) (speed <0.05**m/s)

| One-way analysis of variance |  |
| --- | --- |
| P value | 0.9498 |
| P value summary | ns |
| Are means signif. different? (P < 0.05) | No |

**Table S3. melan-ink4a expressing EGFP-Rab3a.**

Analysis of all speed events.

| Kruskal-Wallis test |  |  |  |
| --- | --- | --- | --- |
| P value | < 0.0001 |  |  |
| Exact or approximate P value? | Gaussian Approximation |  |  |
| P value summary | **** |  |  |
| Do the medians vary signif. (P < 0.05) | Yes |  |  |
| Number of groups | 10 |  |  |
| Kruskal-Wallis statistic | 48.01 |  |  |
|  |  |  |  |
| Dunn's Multiple Comparison Test | Difference in rank sum | Significant? P < 0.05? | Summary |
| 0-10 vs 10-20 | -792.4 | Yes | * |
| 0-10 vs 20-30 | -840.6 | Yes | * |
| 0-10 vs 40-50 | -860.9 | Yes | * |
| 10-20 vs 80-90 | 856.7 | Yes | * |
| 10-20 vs 90-100 | 980.5 | Yes | ** |
| 20-30 vs 80-90 | 904.9 | Yes | ** |
| 20-30 vs 90-100 | 1029 | Yes | *** |
| 30-40 vs 90-100 | 801.4 | Yes | * |
| 40-50 vs 80-90 | 925.3 | Yes | ** |
| 40-50 vs 90-100 | 1049 | Yes | *** |
| 50-60 vs 90-100 | 855.5 | Yes | * |

**Analysis of motile events (speed >0.05μm/s)**

| Kruskal-Wallis test |  |
| --- | --- |
| P value | 0.1756 |
| Exact or approximate P value? | Gaussian Approximation |
| P value summary | ns |
| Do the medians vary signif. (P < 0.05) | No |
| Number of groups | 10 |
| Kruskal-Wallis statistic | 12.72 |

**Analysis of docked events (%) (speed <0.05**m/s)

| One-way analysis of variance |  |
| --- | --- |
| P value | 0.9935 |
| P value summary | ns |
| Are means signif. different? (P < 0.05) | No |

**Table S4. melan-ink4a expressing EGFP-Rab32 and mRFP-Rab27a**

Analysis of all speed events.

| Kruskal-Wallis test |  |  |  |
| --- | --- | --- | --- |
| P value | < 0.0001 |  |  |
| Exact or approximate P value? | Gaussian Approximation |  |  |
| P value summary | **** |  |  |
| Do the medians vary signif. (P < 0.05) | Yes |  |  |
| Number of groups | 10 |  |  |
| Kruskal-Wallis statistic | 306.5 |  |  |
|  |  |  |  |
| Dunn's Multiple Comparison Test | Difference in rank sum | Significant? P < 0.05? | Summary |
| 0-10 vs 60-70 | -484.8 | Yes | *** |
| 0-10 vs 70-80 | -661.1 | Yes | *** |
| 0-10 vs 80-90 | -928.4 | Yes | *** |
| 0-10 vs 90-100 | -1651 | Yes | *** |
| 10-20 vs 80-90 | -625.1 | Yes | *** |
| 10-20 vs 90-100 | -1348 | Yes | *** |
| 20-30 vs 70-80 | -372.4 | Yes | * |
| 20-30 vs 80-90 | -639.8 | Yes | *** |
| 20-30 vs 90-100 | -1363 | Yes | *** |
| 30-40 vs 80-90 | -607.4 | Yes | *** |
| 30-40 vs 90-100 | -1330 | Yes | *** |
| 40-50 vs 70-80 | -425.0 | Yes | ** |
| 40-50 vs 80-90 | -692.3 | Yes | *** |
| 40-50 vs 90-100 | -1415 | Yes | *** |
| 50-60 vs 80-90 | -577.2 | Yes | *** |
| 50-60 vs 90-100 | -1300 | Yes | *** |
| 60-70 vs 80-90 | -443.6 | Yes | ** |
| 60-70 vs 90-100 | -1167 | Yes | *** |
| 70-80 vs 90-100 | -990.3 | Yes | *** |
| 80-90 vs 90-100 | -723.0 | Yes | *** |

**Analysis of motile events (speed >0.05μm/s)**

| Kruskal-Wallis test |  |  |  |
| --- | --- | --- | --- |
| P value | < 0.0001 |  |  |
| Exact or approximate P value? | Gaussian Approximation |  |  |
| P value summary | **** |  |  |
| Do the medians vary signif. (P < 0.05) | Yes |  |  |
| Number of groups | 10 |  |  |
| Kruskal-Wallis statistic | 72.99 |  |  |
|  |  |  |  |
| Dunn's Multiple Comparison Test | Difference in rank sum | Significant? P < 0.05? | Summary |
| 0-10 vs 90-100 | -259.0 | Yes | ** |
| 10-20 vs 90-100 | -315.3 | Yes | *** |
| 20-30 vs 90-100 | -252.7 | Yes | ** |
| 30-40 vs 70-80 | -234.7 | Yes | * |
| 30-40 vs 90-100 | -414.4 | Yes | *** |
| 40-50 vs 70-80 | -258.2 | Yes | * |
| 40-50 vs 90-100 | -437.9 | Yes | *** |
| 50-60 vs 90-100 | -324.1 | Yes | *** |
| 80-90 vs 90-100 | -222.9 | Yes | ** |

**Analysis of docked events (%) (speed <0.05**m/s)

| One-way analysis of variance |  |  |  |  |  |
| --- | --- | --- | --- | --- | --- |
| P value | < 0.0001 |  |  |  |  |
| P value summary | **** |  |  |  |  |
| Are means signif. different? (P < 0.05) | Yes |  |  |  |  |
| Number of groups | 10 |  |  |  |  |
| F | 7.868 |  |  |  |  |
| R square | 0.7798 |  |  |  |  |
|  |  |  |  |  |  |
| ANOVA Table | SS | df | MS |  |  |
| Treatment (between columns) | 1913 | 9 | 212.6 |  |  |
| Residual (within columns) | 540.3 | 20 | 27.02 |  |  |
| Total | 2453 | 29 |  |  |  |
|  |  |  |  |  |  |
| Bonferroni's Multiple Comparison Test | Mean Diff. | t | Significant? P < 0.05? | Summary | 95% CI of diff |
| 0-10 vs 80-90 | 16.20 | 3.817 | Yes | * | 0.05545 to 32.35 |
| 0-10 vs 90-100 | 28.79 | 6.785 | Yes | **** | 12.65 to 44.94 |
| 10-20 vs 90-100 | 23.92 | 5.635 | Yes | *** | 7.771 to 40.06 |
| 20-30 vs 90-100 | 23.64 | 5.571 | Yes | *** | 7.496 to 39.79 |
| 30-40 vs 90-100 | 23.38 | 5.510 | Yes | *** | 7.237 to 39.53 |
| 40-50 vs 90-100 | 26.34 | 6.206 | Yes | *** | 10.19 to 42.48 |
| 50-60 vs 90-100 | 24.06 | 5.668 | Yes | *** | 7.911 to 40.20 |
| 60-70 vs 90-100 | 20.83 | 4.909 | Yes | ** | 4.686 to 36.98 |
| 70-80 vs 90-100 | 17.15 | 4.040 | Yes | * | 1.000 to 33.29 |

**Table S5. melan-ln expressing EGFP-Rab27a.**

Analysis of all speed events.

| Kruskal-Wallis test |  |  |  |
| --- | --- | --- | --- |
| P value | 0.0296 |  |  |
| Exact or approximate P value? | Gaussian Approximation |  |  |
| P value summary | * |  |  |
| Do the medians vary signif. (P < 0.05) | Yes |  |  |
| Number of groups | 10 |  |  |
| Kruskal-Wallis statistic | 18.52 |  |  |
|  |  |  |  |
| Dunn's Multiple Comparison Test | Difference in rank sum | Significant? P < 0.05? | Summary |
| 0-10 vs 40-50 | 429.4 | Yes | * |

**Analysis of motile events (speed >0.05μm/s)**

| Kruskal-Wallis test |  |
| --- | --- |
| P value | 0.6070 |
| Exact or approximate P value? | Gaussian Approximation |
| P value summary | ns |
| Do the medians vary signif. (P < 0.05) | No |
| Number of groups | 10 |
| Kruskal-Wallis statistic | 7.290 |

**Analysis of docked events (%) (speed <0.05μm/s)**

| One-way analysis of variance |  |
| --- | --- |
| P value | 0.9966 |
| P value summary | ns |
| Are means signif. different? (P < 0.05) | No |

**melan-ink4a co-expressing EGFP-Mlph and mRFP-Rab27a.**

Analysis of all speed events.

| Kruskal-Wallis test |  |  |  |
| --- | --- | --- | --- |
| P value | < 0.0001 |  |  |
| Exact or approximate P value? | Gaussian Approximation |  |  |
| P value summary | *** |  |  |
| Do the medians vary signif. (P < 0.05) | Yes |  |  |
| Number of groups | 10 |  |  |
| Kruskal-Wallis statistic | 245.1 |  |  |
|  |  |  |  |
| Dunn's Multiple Comparison Test | Difference in rank sum | Significant? P < 0.05? | Summary |
| 0-10 vs 30-40 | 495.0 | Yes | * |
| 0-10 vs 40-50 | 655.8 | Yes | *** |
| 0-10 vs 50-60 | 963.4 | Yes | *** |
| 0-10 vs 60-70 | 1142 | Yes | *** |
| 0-10 vs 70-80 | 1175 | Yes | *** |
| 0-10 vs 80-90 | 1240 | Yes | *** |
| 0-10 vs 90-100 | 1306 | Yes | *** |
| 10-20 vs 40-50 | 548.5 | Yes | ** |
| 10-20 vs 50-60 | 856.2 | Yes | *** |
| 10-20 vs 60-70 | 1034 | Yes | *** |
| 10-20 vs 70-80 | 1067 | Yes | *** |
| 10-20 vs 80-90 | 1133 | Yes | *** |
| 10-20 vs 90-100 | 1198 | Yes | *** |
| 20-30 vs 40-50 | 468.3 | Yes | * |
| 20-30 vs 50-60 | 776.0 | Yes | *** |
| 20-30 vs 60-70 | 954.1 | Yes | *** |
| 20-30 vs 70-80 | 987.1 | Yes | *** |
| 20-30 vs 80-90 | 1052 | Yes | *** |
| 20-30 vs 90-100 | 1118 | Yes | *** |
| 30-40 vs 50-60 | 468.4 | Yes | * |
| 30-40 vs 60-70 | 646.6 | Yes | *** |
| 30-40 vs 70-80 | 679.5 | Yes | *** |
| 30-40 vs 80-90 | 744.9 | Yes | *** |
| 30-40 vs 90-100 | 810.5 | Yes | *** |
| 40-50 vs 60-70 | 485.8 | Yes | * |
| 40-50 vs 70-80 | 518.7 | Yes | ** |
| 40-50 vs 80-90 | 584.1 | Yes | *** |
| 40-50 vs 90-100 | 649.8 | Yes | *** |

**Analysis of motile events (speed >0.05μm/s)**

| Kruskal-Wallis test |  |  |  |
| --- | --- | --- | --- |
| P value | 0.0551 |  |  |
| Exact or approximate P value? | Gaussian Approximation |  |  |
| P value summary | ns |  |  |
| Do the medians vary signif. (P < 0.05) | No |  |  |
| Number of groups | 10 |  |  |
| Kruskal-Wallis statistic | 16.62 |  |  |
|  |  |  |  |
| Dunn's Multiple Comparison Test | Difference in rank sum | Significant? P < 0.05? | Summary |
| 50-60 vs 80-90 | 203.8 | Yes | * |

**Analysis of docked events (%) (speed <0.05μm/s)**

| One-way analysis of variance |  |
| --- | --- |
| P value | 0.1897 |
| P value summary | ns |
| Are means signif. different? (P < 0.05) | No |
| Number of groups | 10 |
| F | 1.510 |
| R square | 0.3117 |

Table S6**. EGFP-Rab27a rescued melan-ash.**

**DMSO.**

Analysis of all speed events.

| Kruskal-Wallis test |  |  |  |
| --- | --- | --- | --- |
| P value | < 0.0001 |  |  |
| Exact or approximate P value? | Gaussian Approximation |  |  |
| P value summary | **** |  |  |
| Do the medians vary signif. (P < 0.05) | Yes |  |  |
| Number of groups | 10 |  |  |
| Kruskal-Wallis statistic | 1028 |  |  |
|  |  |  |  |
| Dunn's Multiple Comparison Test | Difference in rank sum | Significant? P < 0.05? | Summary |
| 0-10 vs 10-20 | 949.3 | Yes | *** |
| 0-10 vs 20-30 | 1975 | Yes | *** |
| 0-10 vs 30-40 | 2619 | Yes | *** |
| 0-10 vs 40-50 | 2709 | Yes | *** |
| 0-10 vs 50-60 | 2920 | Yes | *** |
| 0-10 vs 60-70 | 3475 | Yes | *** |
| 0-10 vs 70-80 | 3320 | Yes | *** |
| 0-10 vs 80-90 | 3412 | Yes | *** |
| 0-10 vs 90-100 | 3507 | Yes | *** |
| 10-20 vs 20-30 | 1026 | Yes | *** |
| 10-20 vs 30-40 | 1669 | Yes | *** |
| 10-20 vs 40-50 | 1760 | Yes | *** |
| 10-20 vs 50-60 | 1970 | Yes | *** |
| 10-20 vs 60-70 | 2525 | Yes | *** |
| 10-20 vs 70-80 | 2370 | Yes | *** |
| 10-20 vs 80-90 | 2463 | Yes | *** |
| 10-20 vs 90-100 | 2557 | Yes | *** |
| 20-30 vs 30-40 | 643.9 | Yes | ** |
| 20-30 vs 40-50 | 734.5 | Yes | *** |
| 20-30 vs 50-60 | 944.6 | Yes | *** |
| 20-30 vs 60-70 | 1500 | Yes | *** |
| 20-30 vs 70-80 | 1345 | Yes | *** |
| 20-30 vs 80-90 | 1438 | Yes | *** |
| 20-30 vs 90-100 | 1532 | Yes | *** |
| 30-40 vs 60-70 | 855.8 | Yes | *** |
| 30-40 vs 70-80 | 700.8 | Yes | *** |
| 30-40 vs 80-90 | 793.6 | Yes | *** |
| 30-40 vs 90-100 | 887.9 | Yes | *** |
| 40-50 vs 60-70 | 765.2 | Yes | *** |
| 40-50 vs 70-80 | 610.2 | Yes | ** |
| 40-50 vs 80-90 | 703.0 | Yes | *** |
| 40-50 vs 90-100 | 797.3 | Yes | *** |
| 50-60 vs 60-70 | 555.1 | Yes | * |
| 50-60 vs 90-100 | 587.2 | Yes | ** |

**Analysis of motile events (speed >0.05μm/s)**

| Kruskal-Wallis test |  |  |  |
| --- | --- | --- | --- |
| P value | < 0.0001 |  |  |
| Exact or approximate P value? | Gaussian Approximation |  |  |
| P value summary | **** |  |  |
| Do the medians vary signif. (P < 0.05) | Yes |  |  |
| Number of groups | 10 |  |  |
| Kruskal-Wallis statistic | 166.6 |  |  |
|  |  |  |  |
| Dunn's Multiple Comparison Test | Difference in rank sum | Significant? P < 0.05? | Summary |
| 0-10 vs 30-40 | 396.3 | Yes | *** |
| 0-10 vs 40-50 | 563.8 | Yes | *** |
| 0-10 vs 50-60 | 713.2 | Yes | *** |
| 0-10 vs 60-70 | 561.2 | Yes | *** |
| 0-10 vs 70-80 | 442.9 | Yes | *** |
| 0-10 vs 80-90 | 640.9 | Yes | *** |
| 0-10 vs 90-100 | 477.8 | Yes | *** |
| 10-20 vs 30-40 | 361.3 | Yes | *** |
| 10-20 vs 40-50 | 528.7 | Yes | *** |
| 10-20 vs 50-60 | 678.1 | Yes | *** |
| 10-20 vs 60-70 | 526.1 | Yes | *** |
| 10-20 vs 70-80 | 407.9 | Yes | *** |
| 10-20 vs 80-90 | 605.9 | Yes | *** |
| 10-20 vs 90-100 | 442.7 | Yes | *** |
| 20-30 vs 40-50 | 334.8 | Yes | ** |
| 20-30 vs 50-60 | 484.3 | Yes | *** |
| 20-30 vs 60-70 | 332.3 | Yes | * |
| 20-30 vs 80-90 | 412.0 | Yes | *** |
| 30-40 vs 50-60 | 316.8 | Yes | * |

**Analysis of docked events (%) (speed <0.05μm/s)**

| One-way analysis of variance |  |  |  |  |  |
| --- | --- | --- | --- | --- | --- |
| P value | 0.0008 |  |  |  |  |
| P value summary | *** |  |  |  |  |
| Are means signif. different? (P < 0.05) | Yes |  |  |  |  |
| Number of groups | 10 |  |  |  |  |
| F | 4.496 |  |  |  |  |
| R square | 0.5742 |  |  |  |  |
|  |  |  |  |  |  |
| ANOVA Table | SS | df | MS |  |  |
| Treatment (between columns) | 4742 | 9 | 526.9 |  |  |
| Residual (within columns) | 3516 | 30 | 117.2 |  |  |
| Total | 8258 | 39 |  |  |  |
|  |  |  |  |  |  |
| Bonferroni's Multiple Comparison Test | Mean Diff. | t | Significant? P < 0.05? | Summary | 95% CI of diff |
| 0-10 vs 40-50 | -27.83 | 3.635 | Yes | * | -55.44 to -0.2182 |
| 0-10 vs 50-60 | -27.63 | 3.609 | Yes | * | -55.24 to -0.02038 |
| 0-10 vs 60-70 | -34.33 | 4.485 | Yes | ** | -61.94 to -6.722 |
| 0-10 vs 70-80 | -33.37 | 4.359 | Yes | ** | -60.98 to -5.759 |
| 0-10 vs 80-90 | -34.26 | 4.475 | Yes | ** | -61.87 to -6.648 |
| 0-10 vs 90-100 | -32.97 | 4.306 | Yes | ** | -60.58 to -5.357 |

**Nocodazole.**

Analysis of all speed events.

| Kruskal-Wallis test |  |  |  |
| --- | --- | --- | --- |
| P value | < 0.0001 |  |  |
| Exact or approximate P value? | Gaussian Approximation |  |  |
| P value summary | **** |  |  |
| Do the medians vary signif. (P < 0.05) | Yes |  |  |
| Number of groups | 10 |  |  |
| Kruskal-Wallis statistic | 66.67 |  |  |
|  |  |  |  |
| Dunn's Multiple Comparison Test | Difference in rank sum | Significant? P < 0.05? | Summary |
| 0-10 vs 10-20 | 391.5 | Yes | * |
| 0-10 vs 20-30 | 552.2 | Yes | *** |
| 0-10 vs 30-40 | 688.8 | Yes | *** |
| 0-10 vs 40-50 | 704.1 | Yes | *** |
| 0-10 vs 50-60 | 576.2 | Yes | *** |
| 0-10 vs 60-70 | 631.5 | Yes | *** |
| 0-10 vs 70-80 | 598.7 | Yes | *** |
| 0-10 vs 80-90 | 777.8 | Yes | *** |
| 0-10 vs 90-100 | 678.0 | Yes | *** |
| 10-20 vs 80-90 | 386.3 | Yes | * |

**Analysis of motile events (speed >0.05μm/s)**

| Kruskal-Wallis test |  |
| --- | --- |
| P value | 0.9962 |
| Exact or approximate P value? | Gaussian Approximation |
| P value summary | ns |
| Do the medians vary signif. (P < 0.05) | No |
| Number of groups | 10 |
| Kruskal-Wallis statistic | 1.615 |

**Analysis of docked events (%) (speed <0.05μm/s)**

| One-way analysis of variance |  |
| --- | --- |
| P value | 0.9779 |
| P value summary | ns |
| Are means signif. different? (P < 0.05) | No |
| Number of groups | 10 |
| F | 0.2627 |
| R square | 0.1057 |

**Cytochalasin D**

Analysis of all speed events.

| Kruskal-Wallis test |  |  |  |
| --- | --- | --- | --- |
| P value | < 0.0001 |  |  |
| Exact or approximate P value? | Gaussian Approximation |  |  |
| P value summary | **** |  |  |
| Do the medians vary signif. (P < 0.05) | Yes |  |  |
| Number of groups | 10 |  |  |
| Kruskal-Wallis statistic | 275.2 |  |  |
|  |  |  |  |
| Dunn's Multiple Comparison Test | Difference in rank sum | Significant? P < 0.05? | Summary |
| 0-10 vs 50-60 | 554.3 | Yes | *** |
| 0-10 vs 60-70 | 859.2 | Yes | *** |
| 0-10 vs 70-80 | 1021 | Yes | *** |
| 0-10 vs 80-90 | 1074 | Yes | *** |
| 0-10 vs 90-100 | 1471 | Yes | *** |
| 10-20 vs 50-60 | 541.0 | Yes | ** |
| 10-20 vs 60-70 | 845.9 | Yes | *** |
| 10-20 vs 70-80 | 1008 | Yes | *** |
| 10-20 vs 80-90 | 1061 | Yes | *** |
| 10-20 vs 90-100 | 1458 | Yes | *** |
| 20-30 vs 60-70 | 536.8 | Yes | ** |
| 20-30 vs 70-80 | 698.9 | Yes | *** |
| 20-30 vs 80-90 | 751.5 | Yes | *** |
| 20-30 vs 90-100 | 1149 | Yes | *** |
| 30-40 vs 60-70 | 573.1 | Yes | *** |
| 30-40 vs 70-80 | 735.2 | Yes | *** |
| 30-40 vs 80-90 | 787.8 | Yes | *** |
| 30-40 vs 90-100 | 1185 | Yes | *** |
| 40-50 vs 60-70 | 624.8 | Yes | *** |
| 40-50 vs 70-80 | 787.0 | Yes | *** |
| 40-50 vs 80-90 | 839.6 | Yes | *** |
| 40-50 vs 90-100 | 1237 | Yes | *** |
| 50-60 vs 70-80 | 467.0 | Yes | * |
| 50-60 vs 80-90 | 519.7 | Yes | ** |
| 50-60 vs 90-100 | 917.1 | Yes | *** |
| 60-70 vs 90-100 | 612.2 | Yes | *** |
| 70-80 vs 90-100 | 450.1 | Yes | * |

**Analysis of motile events (speed >0.05μm/s)**

| Kruskal-Wallis test |  |  |  |
| --- | --- | --- | --- |
| P value | 0.1321 |  |  |
| Exact or approximate P value? | Gaussian Approximation |  |  |
| P value summary | ns |  |  |
| Do the medians vary signif. (P < 0.05) | No |  |  |
| Number of groups | 10 |  |  |
| Kruskal-Wallis statistic | 13.73 |  |  |
|  |  |  |  |
| Dunn's Multiple Comparison Test | Difference in rank sum | Significant? P < 0.05? | Summary |
| 0-10 vs 90-100 | 300.7 | Yes | * |

**Analysis of docked events (%) (speed <0.05μm/s)**

| One-way analysis of variance |  |
| --- | --- |
| P value | 0.4099 |
| P value summary | ns |
| Are means signif. different? (P < 0.05) | No |
| Number of groups | 10 |
| F | 1.063 |
| R square | 0.1931 |

**EGFP-Mlph rescued melan-ln.**

**DMSO**

Analysis of all speed events.

| Kruskal-Wallis test |  |  |  |
| --- | --- | --- | --- |
| P value | < 0.0001 |  |  |
| Exact or approximate P value? | Gaussian Approximation |  |  |
| P value summary | **** |  |  |
| Do the medians vary signif. (P < 0.05) | Yes |  |  |
| Number of groups | 10 |  |  |
| Kruskal-Wallis statistic | 249.6 |  |  |
|  |  |  |  |
| Dunn's Multiple Comparison Test | Difference in rank sum | Significant? P < 0.05? | Summary |
| 0-10 vs 50-60 | 580.3 | Yes | * |
| 0-10 vs 60-70 | 638.4 | Yes | ** |
| 0-10 vs 70-80 | 1330 | Yes | *** |
| 0-10 vs 80-90 | 1521 | Yes | *** |
| 0-10 vs 90-100 | 1628 | Yes | *** |
| 10-20 vs 70-80 | 1113 | Yes | *** |
| 10-20 vs 80-90 | 1303 | Yes | *** |
| 10-20 vs 90-100 | 1411 | Yes | *** |
| 20-30 vs 70-80 | 1199 | Yes | *** |
| 20-30 vs 80-90 | 1389 | Yes | *** |
| 20-30 vs 90-100 | 1497 | Yes | *** |
| 30-40 vs 70-80 | 1191 | Yes | *** |
| 30-40 vs 80-90 | 1382 | Yes | *** |
| 30-40 vs 90-100 | 1489 | Yes | *** |
| 40-50 vs 70-80 | 962.0 | Yes | *** |
| 40-50 vs 80-90 | 1153 | Yes | *** |
| 40-50 vs 90-100 | 1260 | Yes | *** |
| 50-60 vs 70-80 | 750.0 | Yes | *** |
| 50-60 vs 80-90 | 940.5 | Yes | *** |
| 50-60 vs 90-100 | 1048 | Yes | *** |
| 60-70 vs 70-80 | 691.9 | Yes | ** |
| 60-70 vs 80-90 | 882.4 | Yes | *** |
| 60-70 vs 90-100 | 989.8 | Yes | *** |

**Analysis of motile events (speed >0.05**m/s)

| Kruskal-Wallis test |  |  |  |
| --- | --- | --- | --- |
| P value | < 0.0001 |  |  |
| Exact or approximate P value? | Gaussian Approximation |  |  |
| P value summary | **** |  |  |
| Do the medians vary signif. (P < 0.05) | Yes |  |  |
| Number of groups | 10 |  |  |
| Kruskal-Wallis statistic | 76.04 |  |  |
|  |  |  |  |
| Dunn's Multiple Comparison Test | Difference in rank sum | Significant? P < 0.05? | Summary |
| 0-10 vs 50-60 | 378.5 | Yes | * |
| 0-10 vs 60-70 | 365.8 | Yes | * |
| 0-10 vs 70-80 | 597.1 | Yes | *** |
| 0-10 vs 80-90 | 700.5 | Yes | *** |
| 0-10 vs 90-100 | 519.1 | Yes | *** |
| 10-20 vs 70-80 | 473.8 | Yes | *** |
| 10-20 vs 80-90 | 577.1 | Yes | *** |
| 10-20 vs 90-100 | 395.7 | Yes | * |
| 20-30 vs 70-80 | 472.9 | Yes | *** |
| 20-30 vs 80-90 | 576.2 | Yes | *** |
| 20-30 vs 90-100 | 394.8 | Yes | * |
| 30-40 vs 70-80 | 422.7 | Yes | ** |
| 30-40 vs 80-90 | 526.1 | Yes | *** |
| 40-50 vs 80-90 | 372.6 | Yes | * |

**Analysis of docked events (%) (speed <0.05μm/s)**

| One-way analysis of variance |  |  |  |  |  |
| --- | --- | --- | --- | --- | --- |
| P value | 0.0074 |  |  |  |  |
| P value summary | ** |  |  |  |  |
| Are means signif. different? (P < 0.05) | Yes |  |  |  |  |
| Number of groups | 10 |  |  |  |  |
| F | 3.229 |  |  |  |  |
| R square | 0.4920 |  |  |  |  |
|  |  |  |  |  |  |
| ANOVA Table | SS | df | MS |  |  |
| Treatment (between columns) | 1410 | 9 | 156.7 |  |  |
| Residual (within columns) | 1456 | 30 | 48.53 |  |  |
| Total | 2866 | 39 |  |  |  |
|  |  |  |  |  |  |
| Bonferroni's Multiple Comparison Test | Mean Diff. | t | Significant? P < 0.05? | Summary | 95% CI of diff |
| 0-10 vs 10-20 | -2.227 | 0.4521 | No | ns | -19.99 to 15.54 |
| 0-10 vs 20-30 | -2.591 | 0.5261 | No | ns | -20.36 to 15.17 |
| 0-10 vs 30-40 | -1.738 | 0.3528 | No | ns | -19.50 to 16.03 |
| 0-10 vs 40-50 | -4.717 | 0.9575 | No | ns | -22.48 to 13.05 |
| 0-10 vs 50-60 | -6.848 | 1.390 | No | ns | -24.61 to 10.92 |
| 0-10 vs 60-70 | -8.302 | 1.685 | No | ns | -26.07 to 9.464 |
| 0-10 vs 70-80 | -12.96 | 2.630 | No | ns | -30.72 to 4.809 |
| 0-10 vs 80-90 | -15.88 | 3.225 | No | ns | -33.65 to 1.882 |
| 0-10 vs 90-100 | -17.64 | 3.580 | No | ns | -35.40 to 0.1295 |
| 10-20 vs 20-30 | -0.3644 | 0.07397 | No | ns | -18.13 to 17.40 |
| 10-20 vs 30-40 | 0.4893 | 0.09932 | No | ns | -17.28 to 18.26 |
| 10-20 vs 40-50 | -2.490 | 0.5054 | No | ns | -20.26 to 15.28 |
| 10-20 vs 50-60 | -4.621 | 0.9382 | No | ns | -22.39 to 13.14 |
| 10-20 vs 60-70 | -6.075 | 1.233 | No | ns | -23.84 to 11.69 |
| 10-20 vs 70-80 | -10.73 | 2.178 | No | ns | -28.50 to 7.036 |
| 10-20 vs 80-90 | -13.66 | 2.772 | No | ns | -31.42 to 4.109 |
| 10-20 vs 90-100 | -15.41 | 3.128 | No | ns | -33.18 to 2.357 |
| 20-30 vs 30-40 | 0.8536 | 0.1733 | No | ns | -16.91 to 18.62 |
| 20-30 vs 40-50 | -2.125 | 0.4314 | No | ns | -19.89 to 15.64 |
| 20-30 vs 50-60 | -4.257 | 0.8642 | No | ns | -22.02 to 13.51 |
| 20-30 vs 60-70 | -5.711 | 1.159 | No | ns | -23.48 to 12.06 |
| 20-30 vs 70-80 | -10.37 | 2.104 | No | ns | -28.13 to 7.401 |
| 20-30 vs 80-90 | -13.29 | 2.698 | No | ns | -31.06 to 4.474 |
| 20-30 vs 90-100 | -15.05 | 3.054 | No | ns | -32.81 to 2.721 |
| 30-40 vs 40-50 | -2.979 | 0.6047 | No | ns | -20.74 to 14.79 |
| 30-40 vs 50-60 | -5.111 | 1.038 | No | ns | -22.88 to 12.66 |
| 30-40 vs 60-70 | -6.564 | 1.333 | No | ns | -24.33 to 11.20 |
| 30-40 vs 70-80 | -11.22 | 2.278 | No | ns | -28.98 to 6.547 |
| 30-40 vs 80-90 | -14.15 | 2.872 | No | ns | -31.91 to 3.620 |
| 30-40 vs 90-100 | -15.90 | 3.228 | No | ns | -33.66 to 1.867 |
| 40-50 vs 50-60 | -2.132 | 0.4328 | No | ns | -19.90 to 15.63 |
| 40-50 vs 60-70 | -3.586 | 0.7279 | No | ns | -21.35 to 14.18 |
| 40-50 vs 70-80 | -8.240 | 1.673 | No | ns | -26.01 to 9.526 |
| 40-50 vs 80-90 | -11.17 | 2.267 | No | ns | -28.93 to 6.599 |
| 40-50 vs 90-100 | -12.92 | 2.623 | No | ns | -30.69 to 4.846 |
| 50-60 vs 60-70 | -1.454 | 0.2951 | No | ns | -19.22 to 16.31 |
| 50-60 vs 70-80 | -6.108 | 1.240 | No | ns | -23.87 to 11.66 |
| 50-60 vs 80-90 | -9.035 | 1.834 | No | ns | -26.80 to 8.731 |
| 50-60 vs 90-100 | -10.79 | 2.190 | No | ns | -28.55 to 6.978 |
| 60-70 vs 70-80 | -4.654 | 0.9449 | No | ns | -22.42 to 13.11 |
| 60-70 vs 80-90 | -7.581 | 1.539 | No | ns | -25.35 to 10.18 |
| 60-70 vs 90-100 | -9.334 | 1.895 | No | ns | -27.10 to 8.432 |
| 70-80 vs 80-90 | -2.927 | 0.5942 | No | ns | -20.69 to 14.84 |
| 70-80 vs 90-100 | -4.680 | 0.9501 | No | ns | -22.45 to 13.09 |
| 80-90 vs 90-100 | -1.753 | 0.3558 | No | ns | -19.52 to 16.01 |

**Nocodazole.**

Analysis of all speed events.

| Kruskal-Wallis test |  |  |  |
| --- | --- | --- | --- |
| P value | < 0.0001 |  |  |
| Exact or approximate P value? | Gaussian Approximation |  |  |
| P value summary | **** |  |  |
| Do the medians vary signif. (P < 0.05) | Yes |  |  |
| Number of groups | 10 |  |  |
| Kruskal-Wallis statistic | 114.6 |  |  |
|  |  |  |  |
| Dunn's Multiple Comparison Test | Difference in rank sum | Significant? P < 0.05? | Summary |
| 0-10 vs 50-60 | 517.6 | Yes | * |
| 0-10 vs 70-80 | 605.3 | Yes | ** |
| 0-10 vs 80-90 | 1009 | Yes | *** |
| 0-10 vs 90-100 | 1240 | Yes | *** |
| 10-20 vs 80-90 | 641.5 | Yes | ** |
| 10-20 vs 90-100 | 872.3 | Yes | *** |
| 20-30 vs 80-90 | 829.0 | Yes | *** |
| 20-30 vs 90-100 | 1060 | Yes | *** |
| 30-40 vs 80-90 | 748.1 | Yes | *** |
| 30-40 vs 90-100 | 978.9 | Yes | *** |
| 40-50 vs 80-90 | 792.2 | Yes | *** |
| 40-50 vs 90-100 | 1023 | Yes | *** |
| 50-60 vs 90-100 | 722.0 | Yes | *** |
| 60-70 vs 80-90 | 533.2 | Yes | * |
| 60-70 vs 90-100 | 764.0 | Yes | *** |
| 70-80 vs 90-100 | 634.3 | Yes | ** |

**Analysis of motile events (speed >0.05**m/s)

| Kruskal-Wallis test |  |
| --- | --- |
| P value | 0.2418 |
| Exact or approximate P value? | Gaussian Approximation |
| P value summary | ns |
| Do the medians vary signif. (P < 0.05) | No |
| Number of groups | 10 |
| Kruskal-Wallis statistic | 11.52 |

**Analysis of docked events (%) (speed <0.05**m/s)

| One-way analysis of variance |  |
| --- | --- |
| P value | 0.3183 |
| P value summary | ns |
| Are means signif. different? (P < 0.05) | No |

**Figure S1.**

**A-B melan-ink4a expressing EGFP-Rab27a.**

Analysis of all speed events.

| Kruskal-Wallis test |  |  |  |
| --- | --- | --- | --- |
| P value | < 0.0001 |  |  |
| Exact or approximate P value? | Gaussian Approximation |  |  |
| P value summary | **** |  |  |
| Do the medians vary signif. (P < 0.05) | Yes |  |  |
| Number of groups | 10 |  |  |
| Kruskal-Wallis statistic | 320.6 |  |  |
|  |  |  |  |
| Dunn's Multiple Comparison Test | Difference in rank sum | Significant? P < 0.05? | Summary |
| 0-10 vs 10-20 | 982.6 | Yes | *** |
| 0-10 vs 20-30 | 1751 | Yes | *** |
| 0-10 vs 30-40 | 2437 | Yes | *** |
| 0-10 vs 40-50 | 2591 | Yes | *** |
| 0-10 vs 50-60 | 2626 | Yes | *** |
| 0-10 vs 60-70 | 2759 | Yes | *** |
| 0-10 vs 70-80 | 2360 | Yes | *** |
| 0-10 vs 80-90 | 2356 | Yes | *** |
| 0-10 vs 90-100 | 2140 | Yes | *** |
| 10-20 vs 20-30 | 768.3 | Yes | ** |
| 10-20 vs 30-40 | 1454 | Yes | *** |
| 10-20 vs 40-50 | 1609 | Yes | *** |
| 10-20 vs 50-60 | 1644 | Yes | *** |
| 10-20 vs 60-70 | 1776 | Yes | *** |
| 10-20 vs 70-80 | 1377 | Yes | *** |
| 10-20 vs 80-90 | 1373 | Yes | *** |
| 10-20 vs 90-100 | 1158 | Yes | *** |
| 20-30 vs 30-40 | 685.6 | Yes | * |
| 20-30 vs 40-50 | 840.3 | Yes | ** |
| 20-30 vs 50-60 | 875.5 | Yes | ** |
| 20-30 vs 60-70 | 1008 | Yes | *** |

**Analysis of motile events (speed >0.05**m/s)

| Kruskal-Wallis test |  |  |  |
| --- | --- | --- | --- |
| P value | < 0.0001 |  |  |
| Exact or approximate P value? | Gaussian Approximation |  |  |
| P value summary | **** |  |  |
| Do the medians vary signif. (P < 0.05) | Yes |  |  |
| Number of groups | 10 |  |  |
| Kruskal-Wallis statistic | 65.79 |  |  |
|  |  |  |  |
| Dunn's Multiple Comparison Test | Difference in rank sum | Significant? P < 0.05? | Summary |
| 0-10 vs 10-20 | 333.7 | Yes | ** |
| 0-10 vs 30-40 | 489.1 | Yes | *** |
| 0-10 vs 50-60 | 633.4 | Yes | *** |
| 0-10 vs 60-70 | 594.3 | Yes | *** |
| 0-10 vs 70-80 | 411.0 | Yes | ** |
| 0-10 vs 80-90 | 336.1 | Yes | * |
| 0-10 vs 90-100 | 358.5 | Yes | ** |
| 20-30 vs 50-60 | 481.3 | Yes | *** |
| 20-30 vs 60-70 | 442.1 | Yes | ** |

**Analysis of docked events (%) (speed <0.05**m/s)

| One-way analysis of variance |  |
| --- | --- |
| P value | 0.2177 |
| P value summary | ns |
| Are means signif. different? (P < 0.05) | No |

**C-D EGFP-Rab27b rescued melan-ash.**

Analysis of all speed events.

| Kruskal-Wallis test |  |  |  |
| --- | --- | --- | --- |
| P value | < 0.0001 |  |  |
| Exact or approximate P value? | Gaussian Approximation |  |  |
| P value summary | **** |  |  |
| Do the medians vary signif. (P < 0.05) | Yes |  |  |
| Number of groups | 10 |  |  |
| Kruskal-Wallis statistic | 515.2 |  |  |
|  |  |  |  |
| Dunn's Multiple Comparison Test | Difference in rank sum | Significant? P < 0.05? | Summary |
| 0-10 vs 10-20 | 1557 | Yes | *** |
| 0-10 vs 20-30 | 1521 | Yes | *** |
| 0-10 vs 30-40 | 1904 | Yes | *** |
| 0-10 vs 40-50 | 2188 | Yes | *** |
| 0-10 vs 50-60 | 2435 | Yes | *** |
| 0-10 vs 60-70 | 2772 | Yes | *** |
| 0-10 vs 70-80 | 2827 | Yes | *** |
| 0-10 vs 80-90 | 2897 | Yes | *** |
| 0-10 vs 90-100 | 4096 | Yes | *** |
| 10-20 vs 50-60 | 878.4 | Yes | *** |
| 10-20 vs 60-70 | 1215 | Yes | *** |
| 10-20 vs 70-80 | 1271 | Yes | *** |
| 10-20 vs 80-90 | 1340 | Yes | *** |
| 10-20 vs 90-100 | 2539 | Yes | *** |
| 20-30 vs 40-50 | 666.4 | Yes | * |
| 20-30 vs 50-60 | 913.7 | Yes | *** |
| 20-30 vs 60-70 | 1250 | Yes | *** |
| 20-30 vs 70-80 | 1306 | Yes | *** |
| 20-30 vs 80-90 | 1376 | Yes | *** |
| 20-30 vs 90-100 | 2574 | Yes | *** |
| 30-40 vs 60-70 | 867.8 | Yes | *** |
| 30-40 vs 70-80 | 923.4 | Yes | *** |
| 30-40 vs 80-90 | 993.2 | Yes | *** |
| 30-40 vs 90-100 | 2192 | Yes | *** |
| 40-50 vs 80-90 | 709.3 | Yes | * |
| 40-50 vs 90-100 | 1908 | Yes | *** |
| 50-60 vs 90-100 | 1661 | Yes | *** |
| 60-70 vs 90-100 | 1324 | Yes | *** |
| 70-80 vs 90-100 | 1268 | Yes | *** |
| 80-90 vs 90-100 | 1199 | Yes | *** |

**Analysis of motile events (speed >0.05**m/s)

| Kruskal-Wallis test |  |  |  |
| --- | --- | --- | --- |
| P value | < 0.0001 |  |  |
| Exact or approximate P value? | Gaussian Approximation |  |  |
| P value summary | **** |  |  |
| Do the medians vary signif. (P < 0.05) | Yes |  |  |
| Number of groups | 10 |  |  |
| Kruskal-Wallis statistic | 72.60 |  |  |
|  |  |  |  |
| Dunn's Multiple Comparison Test | Difference in rank sum | Significant? P < 0.05? | Summary |
| 0-10 vs 30-40 | 336.3 | Yes | * |
| 0-10 vs 60-70 | 494.8 | Yes | *** |
| 0-10 vs 70-80 | 534.2 | Yes | *** |
| 0-10 vs 80-90 | 574.9 | Yes | *** |
| 0-10 vs 90-100 | 812.3 | Yes | *** |
| 10-20 vs 90-100 | 538.3 | Yes | *** |
| 20-30 vs 90-100 | 569.2 | Yes | *** |
| 30-40 vs 90-100 | 476.0 | Yes | ** |
| 40-50 vs 90-100 | 504.5 | Yes | ** |
| 50-60 vs 90-100 | 558.0 | Yes | *** |

**Analysis of docked events (%) (speed <0.05**m/s)

| One-way analysis of variance |  |
| --- | --- |
| P value | 0.2034 |
| P value summary | ns |
| Are means signif. different? (P < 0.05) | No |

**E-F melan-ink4a expressing EGFP-Rab27b.**

**Analysis of all speed e**vents.

| Kruskal-Wallis test |  |  |  |
| --- | --- | --- | --- |
| P value | < 0.0001 |  |  |
| Exact or approximate P value? | Gaussian Approximation |  |  |
| P value summary | **** |  |  |
| Do the medians vary signif. (P < 0.05) | Yes |  |  |
| Number of groups | 10 |  |  |
| Kruskal-Wallis statistic | 163.4 |  |  |
|  |  |  |  |
| Dunn's Multiple Comparison Test | Difference in rank sum | Significant? P < 0.05? | Summary |
| 0-10 vs 10-20 | 900.9 | Yes | *** |
| 0-10 vs 20-30 | 894.7 | Yes | *** |
| 0-10 vs 30-40 | 1067 | Yes | *** |
| 0-10 vs 40-50 | 1370 | Yes | *** |
| 0-10 vs 50-60 | 1347 | Yes | *** |
| 0-10 vs 60-70 | 1964 | Yes | *** |
| 0-10 vs 70-80 | 1863 | Yes | *** |
| 0-10 vs 80-90 | 1625 | Yes | *** |
| 0-10 vs 90-100 | 1894 | Yes | *** |
| 10-20 vs 60-70 | 1063 | Yes | *** |
| 10-20 vs 70-80 | 962.4 | Yes | *** |
| 10-20 vs 80-90 | 723.8 | Yes | * |
| 10-20 vs 90-100 | 993.5 | Yes | *** |
| 20-30 vs 60-70 | 1070 | Yes | *** |
| 20-30 vs 70-80 | 968.5 | Yes | *** |
| 20-30 vs 80-90 | 730.0 | Yes | * |
| 20-30 vs 90-100 | 999.7 | Yes | *** |
| 30-40 vs 60-70 | 897.1 | Yes | *** |
| 30-40 vs 70-80 | 796.0 | Yes | ** |
| 30-40 vs 90-100 | 827.2 | Yes | ** |

**Analysis of motile events (speed >0.05**m/s)

| Kruskal-Wallis test |  |  |  |
| --- | --- | --- | --- |
| P value | < 0.0001 |  |  |
| Exact or approximate P value? | Gaussian Approximation |  |  |
| P value summary | **** |  |  |
| Do the medians vary signif. (P < 0.05) | Yes |  |  |
| Number of groups | 10 |  |  |
| Kruskal-Wallis statistic | 34.53 |  |  |
|  |  |  |  |
| Dunn's Multiple Comparison Test | Difference in rank sum | Significant? P < 0.05? | Summary |
| 0-10 vs 40-50 | 394.2 | Yes | ** |
| 0-10 vs 50-60 | 328.4 | Yes | * |
| 0-10 vs 60-70 | 458.8 | Yes | *** |
| 0-10 vs 80-90 | 330.3 | Yes | * |

**Analysis of docked events (%) (speed <0.05**m/s)

| One-way analysis of variance |  |
| --- | --- |
| P value | 0.8194 |
| P value summary | ns |
| Are means signif. different? (P < 0.05) | No |
