# Supplementary figures and images for: Semi-Automated Analysis of Organelle Movement and Membrane Content: Understanding Rab-Motor Complex Transport Function
Source: Traffic. 2011 Oct 11;12(12):1686–701. doi: 10.1111/j.1600-0854.2011.01283.x (PMC3264752; doi:10.1111/j.1600-0854.2011.01283.x)

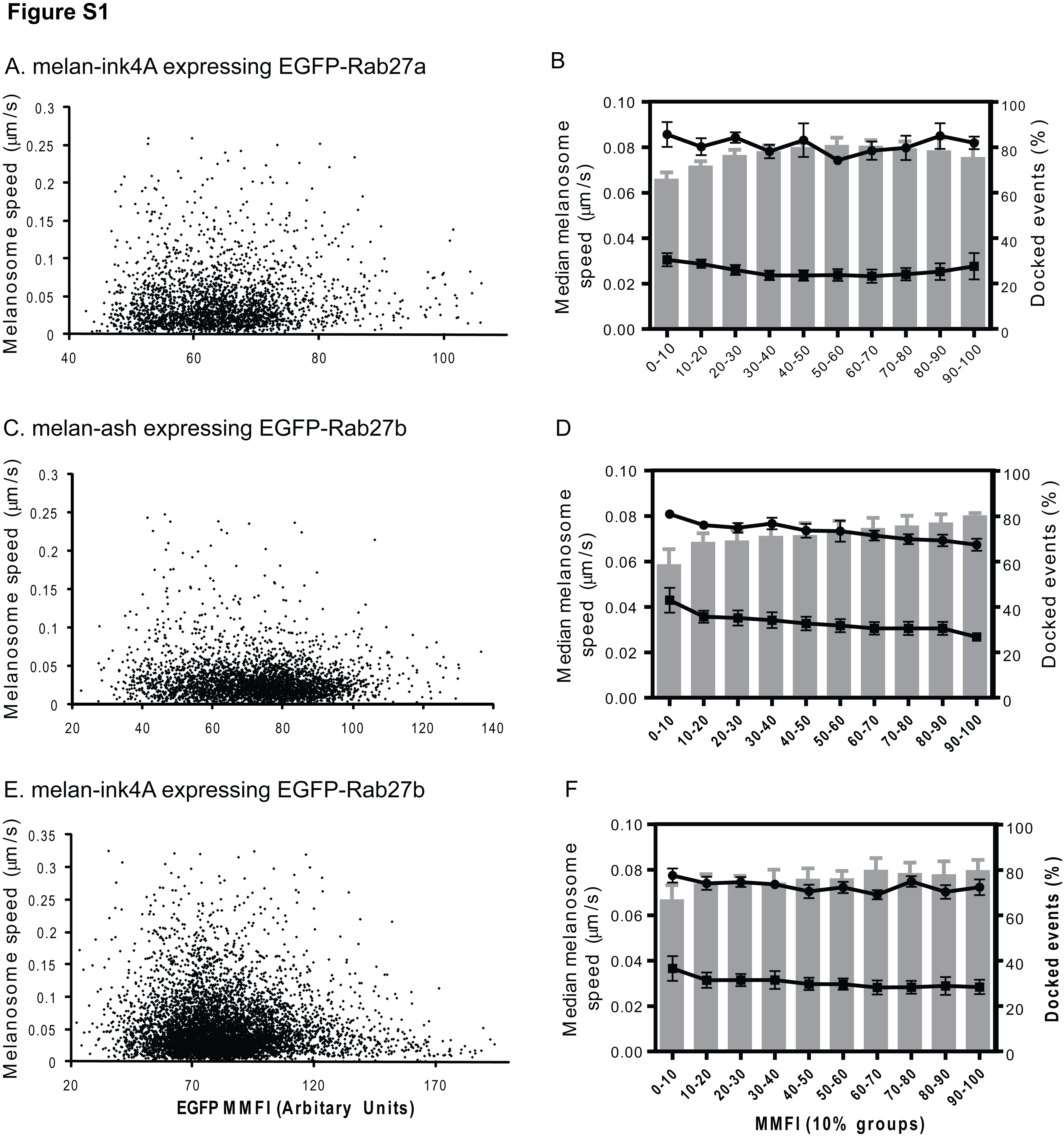

Supplement: Supplementary file 2 [file tra0012-1686-SD2.jpg]

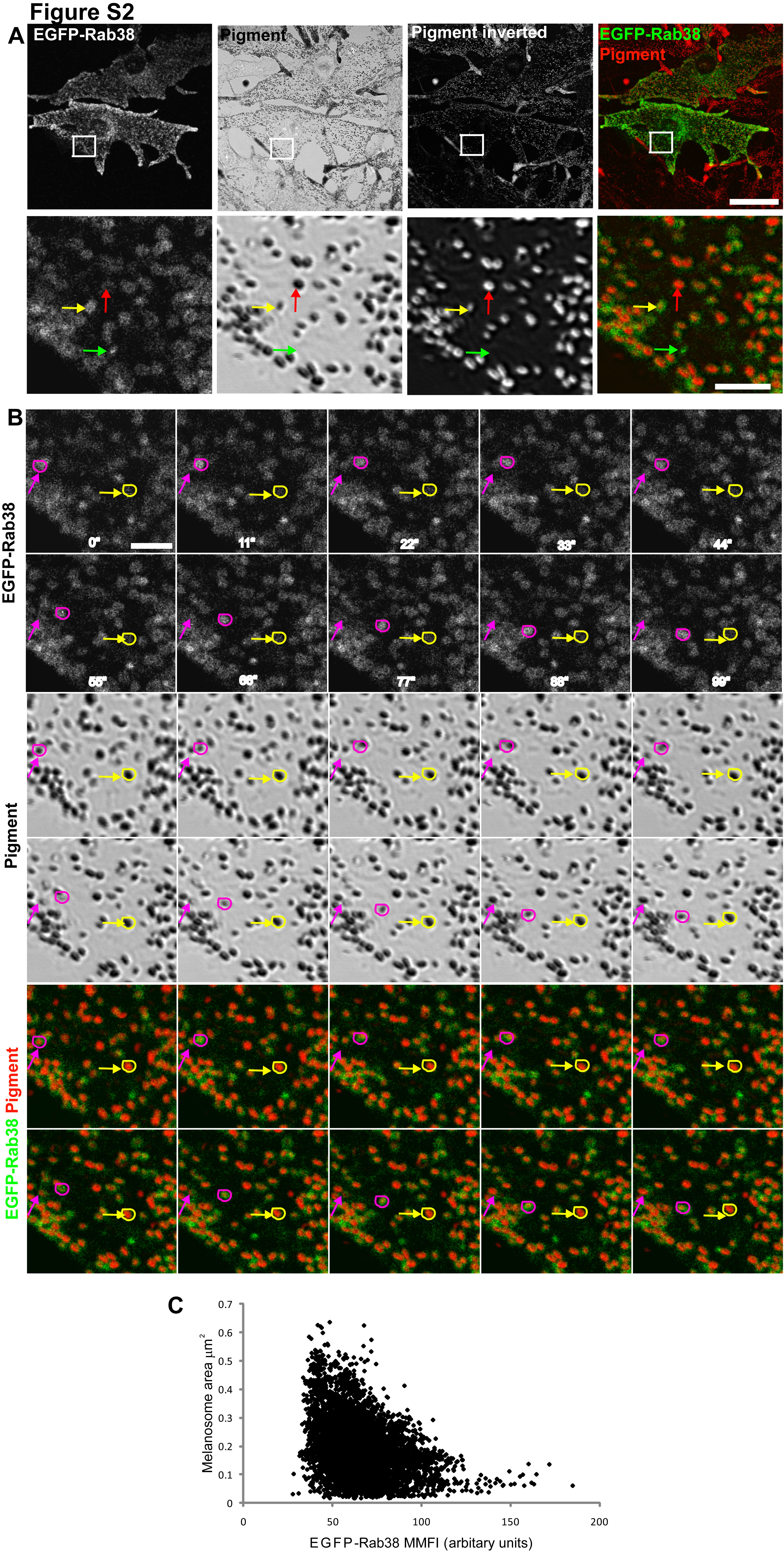

Supplement: Supplementary file 3 [file tra0012-1686-SD3.jpg]

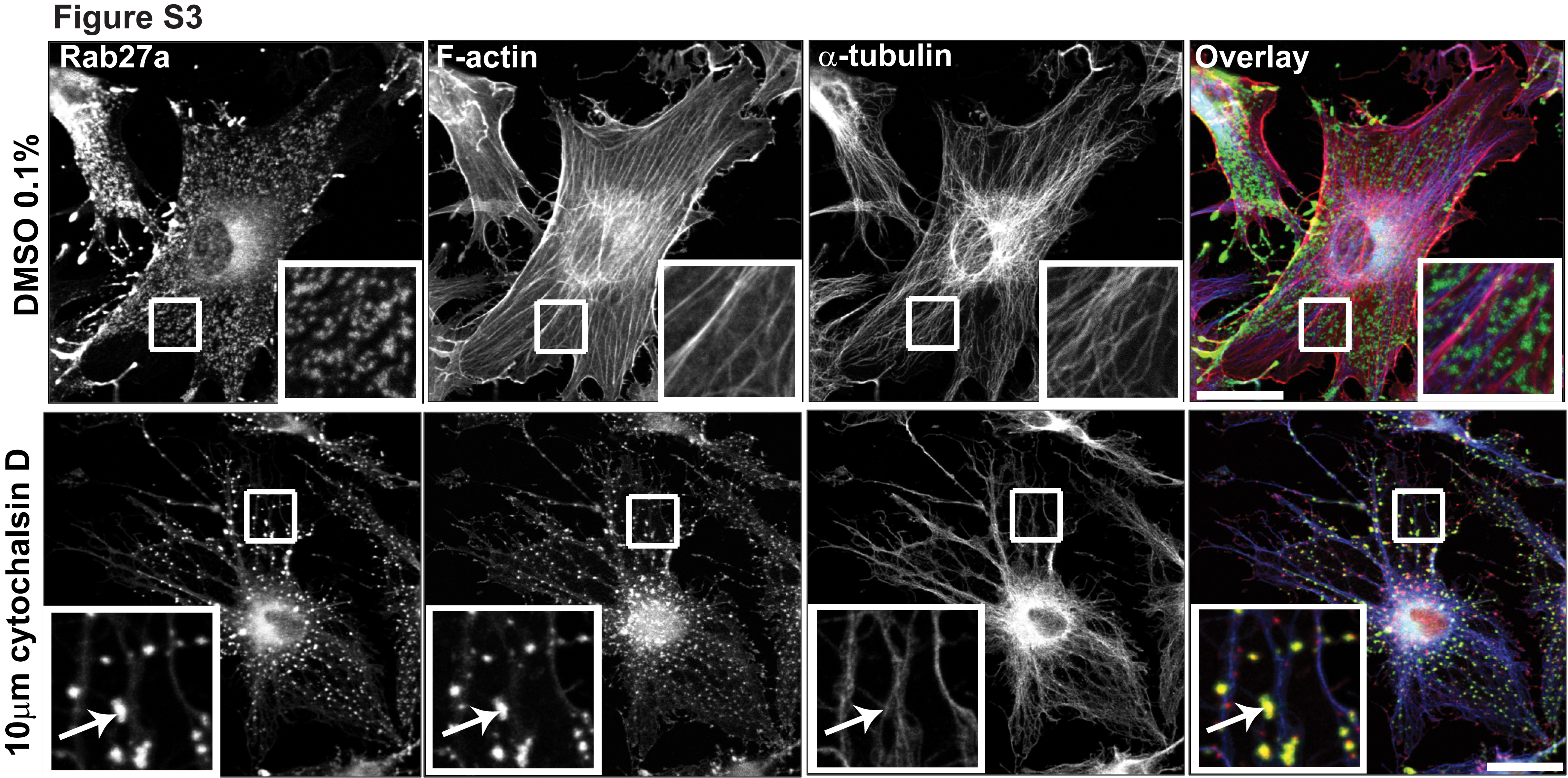

Supplement: Supplementary file 4 [file tra0012-1686-SD4.jpg]
